# Smooth muscle–derived progenitor cell myofibroblast differentiation through KLF4 downregulation promotes arterial remodeling and fibrosis

**DOI:** 10.1172/jci.insight.139445

**Published:** 2020-12-03

**Authors:** Sizhao Lu, Austin J. Jolly, Keith A. Strand, Allison M. Dubner, Marie F. Mutryn, Karen S. Moulton, Raphael A. Nemenoff, Mark W. Majesky, Mary C.M. Weiser-Evans

**Affiliations:** 1 Division of Renal Diseases and Hypertension, Department of Medicine, and; 2 Division of Cardiology, Department of Medicine, and; 3 Consortium for Fibrosis Research and Translation, School of Medicine, University of Colorado Anschutz Medical Campus, Aurora, Colorado, USA.; 4 Center for Developmental Biology and Regenerative Medicine, Seattle Children’s Research Institute, Seattle, Washington, USA.; 5 Department of Pediatrics and Department of Laboratory Medicine and Pathology, University of Washington, Seattle, Washington, USA.; 6 Cardio Vascular Pulmonary Research Lab, University of Colorado Anschutz Medical Campus, Aurora, Colorado, USA.

**Keywords:** Stem cells, Vascular Biology, Adult stem cells, Fibrosis

## Abstract

Resident vascular adventitial SCA1^+^ progenitor (AdvSca1) cells are essential in vascular development and injury. However, the heterogeneity of AdvSca1 cells presents a unique challenge in understanding signaling pathways orchestrating their behavior in homeostasis and injury responses. Using smooth muscle cell (SMC) lineage-tracing models, we identified a subpopulation of AdvSca1 cells (AdvSca1-SM) originating from mature SMCs that undergo reprogramming in situ and exhibit a multipotent phenotype. Here we employed lineage tracing and RNA-sequencing to define the signaling pathways regulating SMC-to-AdvSca1-SM cell reprogramming and AdvSca1-SM progenitor cell phenotype. Unbiased hierarchical clustering revealed that genes related to hedgehog/WNT/beta-catenin signaling were significantly enriched in AdvSca1-SM cells, emphasizing the importance of this signaling axis in the reprogramming event. Leveraging AdvSca1-SM–specific expression of GLI-Kruppel family member GLI1 (*Gli1*), we generated *Gli1*-CreER^T2^-ROSA26-YFP reporter mice to selectively track AdvSca1-SM cells. We demonstrated that physiologically relevant vascular injury or AdvSca1-SM cell–specific Kruppel-like factor 4 (*Klf4*) depletion facilitated the proliferation and differentiation of AdvSca1-SM cells to a profibrotic myofibroblast phenotype rather than macrophages. Surprisingly, AdvSca1-SM cells selectively contributed to adventitial remodeling and fibrosis but little to neointima formation. Together, these findings strongly support therapeutics aimed at preserving the AdvSca1-SM cell phenotype as a viable antifibrotic approach.

## Abstract 2

Smooth muscle cell–derived resident vascular adventitial progenitor cells adopt a myofibroblast phenotype in response to vascular injury and play a dominant role in vascular fibrosis.

## Graphical abstract

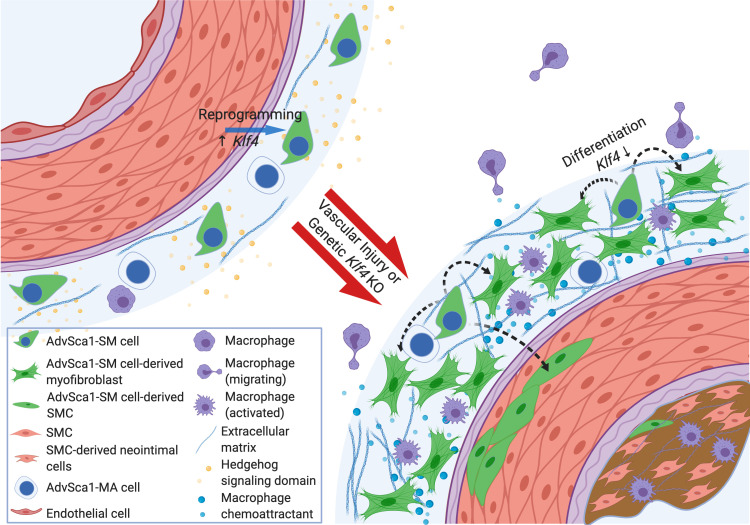

## Introduction

The vessel wall is a complex, multilayered tissue consisting of multiple cell types that dynamically communicate with each other to regulate vessel homeostasis and vascular disease progression. The past several years have seen significant advances in our understanding of the contribution of the adventitia in these settings (1). Rather than a relatively inert tissue that functions to provide structural support, reports over the years have shown that the adventitia is populated by dynamic populations of leukocytes, microvessels, fibroblasts, adipocytes, and resident progenitor cells (1–3). These cell populations not only maintain normal vessel homeostasis but also respond rapidly and robustly to many kinds of vascular injury. The discovery that the normal adventitia is home to resident populations of SCA1^+^ vascular stem/progenitor cells (AdvSca1 cells) with multiple fate potentials raised new and important questions about roles these cells play in growth, remodeling, repair, and disease of the artery wall (4–6). While several subpopulations of AdvSca1 progenitor cells with heterogenous differentiation potentials have been described (7–11), the role of specific subtypes in both normal vessel homeostasis and disease remains unclear.

A central unanswered question concerns what functions AdvSca1 cells actually play during the lifetime of arteries in vivo. Much current speculation has centered on a role for AdvSca1 progenitor cells in neointimal formation after vascular injury. The evidence for such a role centers largely on 2 types of experimental models: (a) a vein graft interposed in the arterial circulation (7, 12) and (b) transection of a conduit artery followed by anastomosis of the severed ends (13, 14). While these models are clinically relevant, they represent the extreme end of the scale of vascular wall injury and are found only after surgical intervention in vivo. In comparative studies that employed less severe forms of arterial injury (13, 14), AdvSca1 progenitor cell responses with respect to neointimal formation were quite different. For example, severe injuries produce neointimas composed, at least in part, of smooth muscle cells (SMCs) derived from adventitial progenitor cells. By contrast, moderate injuries without surgical transection or transplantation where the artery remains intact produce neointimas composed of SMCs derived predominantly from preexisting medial SMCs (15, 16). Additionally, the existence of a distinct SCA1^+^CD45^+^ subpopulation of AdvSca1 myeloid progenitors with hematopoietic potential was demonstrated (11, 17), and in models of heart failure (18) and hypertension (19), data support a role for AdvSca1 cells in coronary artery and aortic perivascular fibrosis, respectively. Collectively, a pathogenic role for AdvSca1 progenitor cells has been implied. However, given the heterogeneity of AdvSca1 cells, the lack of a physiological lineage tracing system to track specific populations of these cells precludes an accurate assessment of the contribution of distinct AdvSca1 cell subtypes to disease progression. Additionally, the fate of specific AdvSca1 cells in moderate, clinically relevant injury models has not been carefully studied.

SMCs are specialized cells that express high levels of SMC-specific contractile proteins (e.g., smooth muscle–specific α-actin, αSMA; smooth muscle–specific myosin heavy chain) necessary for their function in the maintenance of vessel homeostasis, vessel tone, blood pressure, and blood flow distribution (20, 21). Under pathological conditions, however, SMCs are capable of undergoing profound phenotypic and functional changes. Studies on these phenotypic transitions have mostly focused on medial SMC migration toward the intima and their contribution to intimal hyperplasia and inflammation. Our previous reports using a highly specific SMC lineage-mapping approach in the setting of restenosis demonstrated that the majority of proliferating intimal cells derive from mature SMCs (15). Similar findings were demonstrated by others using the same approach in the setting of atherosclerosis (22–24). Little attention was given to the possibility that SMCs migrate and contribute to the composition of other vessel wall layers, notably the adventitia. Our published work using this SMC lineage-mapping approach conclusively demonstrated that mature SMCs migrate into the adventitia, are reprogrammed into a distinct subset of AdvSca1 progenitor cells that we termed AdvSca1-SM cells, and reside in an adventitial progenitor niche (25). AdvSca1-SM cells exhibit a multipotent phenotype marked by their ability to differentiate into multiple cell types in response to specific environmental cues, including SMCs, endothelial cells, macrophages, myofibroblasts, and adipocytes. The ability of mature SMCs to reprogram in vivo has important implications as this represents a novel mechanism whereby SMCs maintain and replenish a subpopulation of resident progenitor cells and, likewise, AdvSca1-SM cells replenish the medial SMC pool, contributing to vessel wall repair. Importantly, given their multipotent phenotype, these cells are uniquely poised to rapidly respond to vascular injury and contribute to disease progression and pathological vascular remodeling.

We previously demonstrated that induction of the pluripotency-associated transcription factor, Kruppel-like factor 4 (*Klf4*), is essential for SMC reprogramming to AdvSca1-SM cells (25), and others have shown that *Klf4* induction is essential for SMC transitions in the setting of atherosclerotic lesion progression (23) and cancer progression (26). However, underlying molecular mechanisms of induction and mechanisms regulating AdvSca1-SM cell phenotype maintenance remain unknown. In addition, the fate of AdvSca1-SM cells in the setting of restenosis is unclear. The focus of the studies described here was to use RNA-Seq, cell fate tracking, and a moderate injury model to better understand the normal fate of AdvSca1-SM cells in vivo. We report here that SMC-derived AdvSca1-SM progenitor cells selectively exhibited constitutive expression of a hedgehog/WNT/KLF4 signaling axis associated with a progenitor cell phenotype. In response to carotid artery ligation injury, however, AdvSca1-SM cells downregulated KLF4 and, subsequently, a progenitor cell gene signature and predominantly adopted a myofibroblast phenotype. Some AdvSca1-SM–derived cells repaired the medial wall, but few moved into the intima and formed intimal SMCs. In this setting, using a physiologically relevant fate-mapping approach, AdvSca1-SM–derived myofibroblasts exhibited a robust fibrotic response and remodeled the vessel wall to become a stiffer and less compliant artery, which increases the risk for development of hypertension and atherosclerotic vascular disease.

## Results

### Global analysis of genes differentially expressed between mature SMCs, SMC-derived AdvSca1-SM cells, and non–SMC-derived AdvSca1-MA cells.

**Figure 1:**
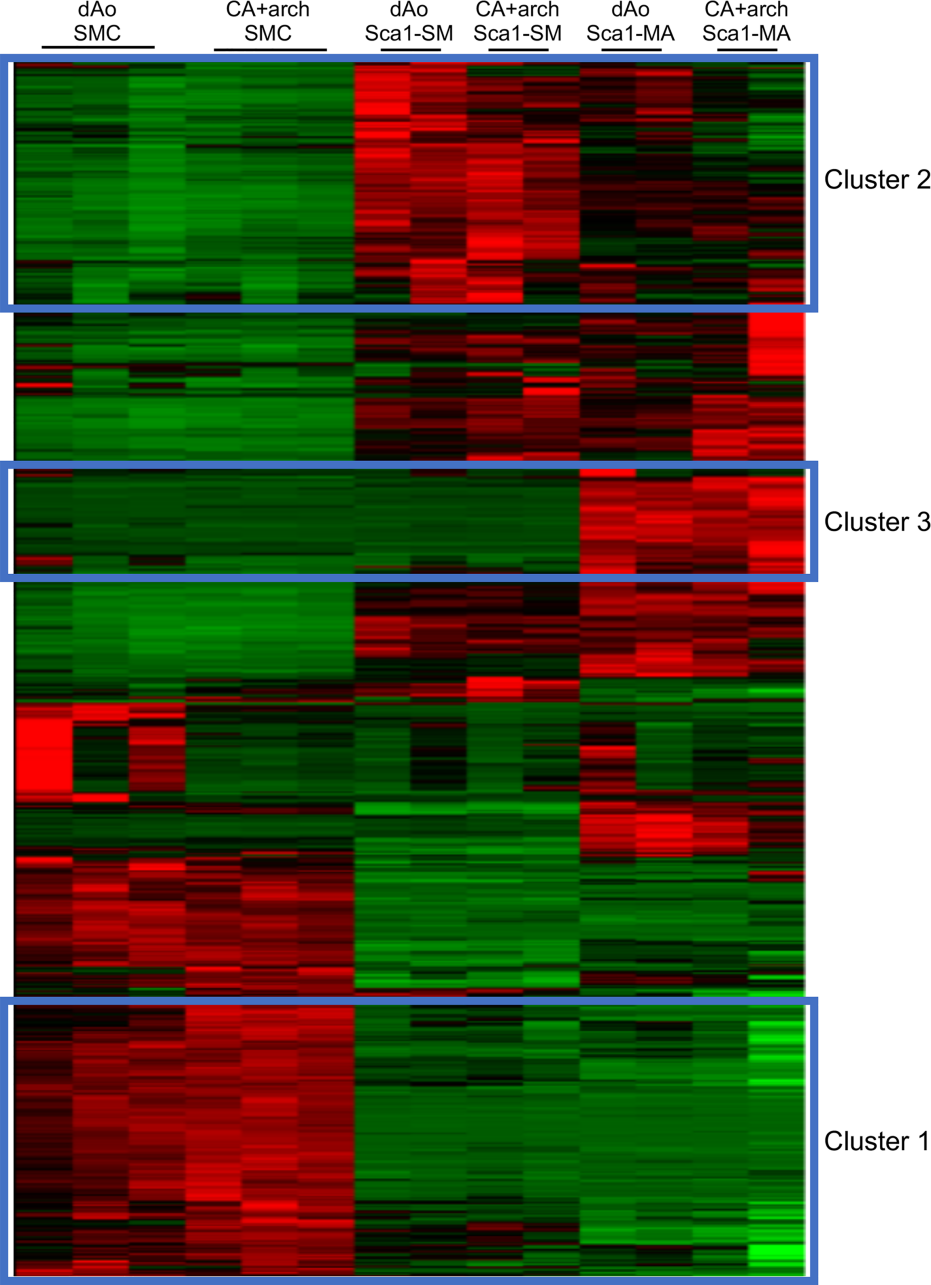
Global analysis of genes differentially expressed between mature SMCs, SMC-derived AdvSca1-SM cells, and non–SMC-derived AdvSca1-MA cells. Mature SMCs, AdvSca1-SM cells, and AdvSca1-MA cells were recovered from the carotid artery + aortic arch (CA+arch) and descending aortae (dAo) of SMC reporter mice as described in Methods. Total RNA was isolated from cell populations from pooled, digested arteries and analyzed by RNA-Seq. *N* = 3 independent experiments using arteries from 10–12 pooled mice per experiment were used for analysis. Differentially expressed genes were identified in all pairwise comparisons between the recovered populations (5265 genes). Hierarchical clustering was performed on the set of 5265 genes and clusters with high expression in specific populations were identified: cluster 1 — genes highly and selectively expressed in mature SMCs; cluster 2 — genes highly and selectively expressed in SMC-derived AdvSca1-SM cells; cluster 3 — genes highly expressed in non–SMC-derived AdvSca1-MA cells. Red, upregulated genes; green, downregulated genes.

Using 2 highly specific SMC lineage-mapping approaches combined with analysis of a retained SMC-specific epigenetic lineage mark, our previous report demonstrated that mature SMCs move into the adventitia, are reprogrammed into a subset of AdvSca1 progenitor cells (AdvSca1-SM cells), and reside in an adventitial progenitor niche in close association with another distinct subset of AdvSca1 progenitor cells (AdvSca1-MA cells) (25). While we demonstrated that induction of the pluripotency-associated transcription gene, *Klf4*, is essential for SMC reprogramming, the underlying molecular mechanism of induction remains unknown. To gain further insight into the mechanisms of SMC reprogramming and the functional properties of distinct AdvSca1 cell populations, we performed transcriptome profiling using RNA-Seq. We used flow cytometry–based sorting to recover cells from the carotid artery + aortic arch and descending aorta of SMC reporter mice (25) as described in Methods (Supplemental Figure 1A and B; supplemental material available online with this article; https://doi.org/10.1172/jci.insight.139445DS1). Cells were sorted based on endogenous yellow fluorescence protein (YFP) expression (SMC origin) and SCA1 expression to recover mature SMCs (YFP^+^SCA1^–^), SMC-derived AdvSca1-SM cells (YFP^+^SCA1^+^), and non–SMC-derived AdvSca1-MA cells (YFP^–^SCA1^+^). RNA was extracted from 3 separate isolations of cells, with arteries from 10–12 mice contributing to each isolation, and subjected to RNA-Seq analysis; sufficient high-quality RNA was isolated to sequence triplicate samples of mature SMCs and duplicate samples of AdvSca1-SM and AdvSca1-MA cells. We analyzed differential gene expression between these populations, and their transcriptional profiles were compared to published databases. In a pairwise analysis of differentially expressed genes, a differentially expressed gene was expected to have expression level fragments per kilobase of exon per million fragments mapped (FPKM) of more than 1 in at least 1 condition and have more than 2-fold change in expression with a false discovery rate–adjusted *P* value less than 0.05 between the 2 conditions. Among the 3 cell populations, we identified 5265 genes that were differentially expressed. Using unbiased hierarchical clustering of all 5265 genes to identify relationships among cell populations, we identified several distinct gene groups (Figure 1). Analysis indicated that cell replicates clustered together. Pathway overrepresentation analysis of the gene clusters was conducted using Web-based bioinformatics tools at ConsensusPathDB (27), searching in the Kyoto Encyclopedia of Genes and Genomes (KEGG) and Reactome databases. Several characteristic pathway signatures of individual clusters were identified, including those overrepresented in mature SMCs compared with the AdvSca1 cell populations (cluster 1; Figure 1), those overrepresented in AdvSca1-SM cells compared with the other cell populations (cluster 2; Figure 1), and those overrepresented in AdvSca1-MA cells compared with mature SMCs or AdvSca1-SM cells (cluster 3; Figure 1). Characteristic pathway signatures of individual clusters are discussed in greater detail below.

### SMC-specific genes are selectively overrepresented in mature SMCs.

In cluster 1, pathway overrepresentation analysis highly ranked multiple gene sets related to muscle contraction and, in particular, SMC contraction (Supplemental Figure 1C). Gene Set Enrichment Analysis (GSEA) (28) was also conducted to determine if a defined set of genes showed statistical significance among populations of cells. Compared with AdvSca1-SM and AdvSca1-MA cells, in agreement with the pathway overrepresentation analysis, genes related to SMC contraction and phenotype were significantly enriched in mature SMCs, as expected and similar to our published data (25) (Supplemental Figure 1D); these were also identified in cluster 1 (Supplemental Figure 1E) by pathway analysis. In contrast, and in agreement with our previous findings (25), stemness-related genes were found to be overrepresented in AdvSca1-SM and AdvSca1-MA cells (e.g., *Ly6a*, *Cd34*, *Cd44*, *Klf4*, *Myc*; data not shown). These results demonstrate the robustness and accuracy of our RNA-Seq data set.

### AdvSca1-SM cells express 2 major classes of genes: genes related to hedgehog/WNT/beta-catenin signaling and extracellular matrix and matrix-modifying genes.

**Figure 2:**
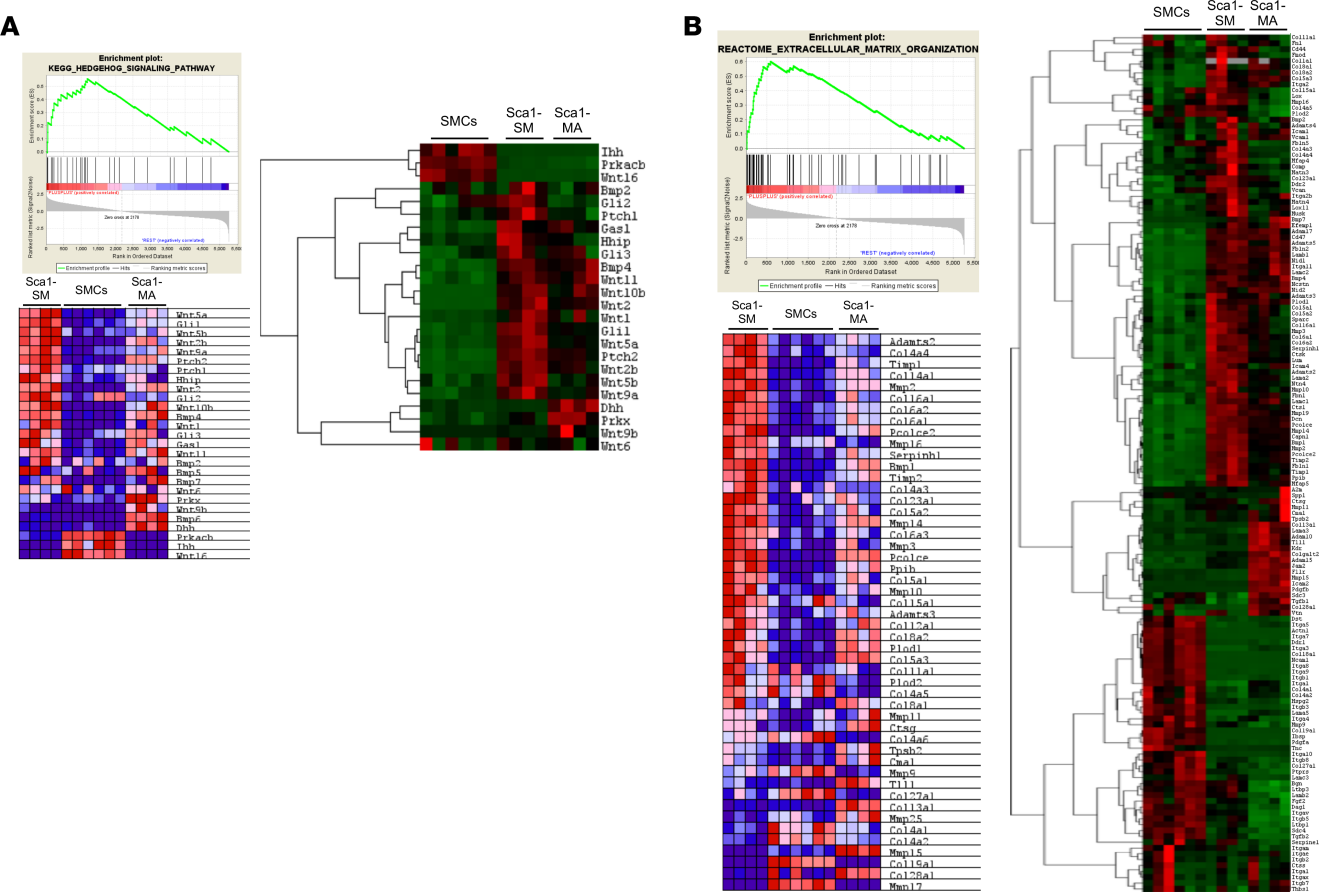
AdvSca1-SM cells express 2 major classes of genes: genes related to hedgehog/WNT/beta-catenin signaling and ECM and ECM-modifying genes. (**A**) Left: GSEA enrichment plot (top) and KEGG hedgehog signaling heatmap (bottom). GSEA tool was used to examine gene enrichment in AdvSca1-SM cells compared with mature SMCs and AdvSca1-MA cells. Right: Heatmap of levels of differentially expressed genes related to hedgehog and WNT signaling. Gene list was based on genes in the KEGG hedgehog signaling pathway (italicized pathway in Table 1). (**B**) Left: GSEA enrichment plot (top) and Reactome extracellular matrix organization heatmap (bottom). GSEA tool was used to examine gene enrichment in AdvSca1-SM cells compared with mature SMCs and AdvSca1-MA cells. Right: Heatmap of levels of differentially expressed genes related to ECM and ECM remodeling. Gene list was based on consensus of genes in the top-ranking ECM-related pathways (Reactome: extracellular matrix organization, collagen formation, degradation of the extracellular matrix, and collagen biosynthesis and modifying enzymes; bold pathways in Table 1). For GSEA heatmaps, red indicates upregulated genes, and blue indicates downregulated genes. For pathway analysis heatmaps, red indicates upregulated genes, and green indicates downregulated genes.

As indicated by pathway overrepresentation analysis, cluster 2 (genes highly expressed in SMC-derived AdvSca1-SM cells) was enriched in 2 major pathways: genes associated with hedgehog/WNT/beta-catenin signaling and, interestingly, genes related to extracellular matrix (ECM) and ECM-modifying genes (Table 1). Complete GSEA comparing AdvSca1-SM cells to both SMCs and AdvSca1-MA cells resulted in 9 of the top 20 “hits” being related to ECM or SHH/WNT signaling (1 — Reactome ECM organization, 3 — PID Hedgehog pathway, 5 — KEGG Glycosaminoglycan degradation, 7 — Naba Core Matrisome, 11 — KEGG Hedgehog signaling, 13— Reactome Collagen formation, 16 — Naba ECM glycoproteins, 17 — Naba ECM regulators, 18 — Naba other glycan degradation, 20 — PID WNT signaling; data not shown). Heatmaps (Figure 2A) demonstrated that the majority of genes in the hedgehog signaling pathway, such as GLI-Kruppel family members *Gli1* and *Gli2*, patched 1 and 2 (*Ptch1* and *Ptch2*), smoothened frizzled class receptor (*Smo*), hedgehog-interacting protein (*Hhip*), and several *Wnt* family members, were upregulated in AdvSca1-SM cells compared with mature SMCs or AdvSca1-MA cells. Hedgehog/WNT signaling pathways are key regulators of adult stem cell regulation (29, 30), suggesting that SMC reprogramming and AdvSca1-SM cell maintenance involve induction of this signaling axis. In addition to hedgehog/WNT signaling, GSEA (Figure 2B, left) and pathway analysis (Figure 2B, right) demonstrated that ECM and ECM-modifying genes, such as *Col1a1*, *Col3a1*, *Vcan1*, *Lox*, *Pcolce*, and *Plod2*, were highly expressed in AdvSca1-SM cells compared with mature SMCs and AdvSca1-MA cells, suggesting that AdvSca1-SM cells are major contributors to the surrounding microenvironmental vascular matrix that regulates the properties of adventitial cells. RNA was extracted from mature SMCs, AdvSca1-SM cells, and AdvSca1-MA cells from 3 independent isolations of arteries from 10–12 mice per isolation, and quantitative PCR (qPCR) was used to validate overexpression of a subset of the identified genes from the RNA-Seq data set selectively in AdvSca1-SM cells (Supplemental Figure 2).

### Non–SMC-derived AdvSca1-MA cells express a tissue-resident endothelial stem cell gene signature.

Cluster 3 (Figure 1), genes highly expressed by AdvSca1-MA cells, was enriched with genes related to Notch signaling, NO pathway, cell-cell junction organization, and VEGF signaling (pathway analysis not shown). A recent manuscript demonstrated that bone marrow stromal cell antigen 1/CD157 (*Bst1*/CD157) is highly expressed in functional resident vascular endothelial stem cells (VESCs) that reside in large arteries and veins of a number of organs, including the heart, lung, and liver as well as the aortic adventitia (31). Because *Bst1* was also found to be upregulated in AdvSca1-MA cells compared with AdvSca1-SM cells and mature SMCs, to determine the similarity of AdvSca1-MA cells to these previously described VESCs, we compared the 5265 differentially expressed genes generated from our data set with the top 500 upregulated genes in the VESC population from this study and generated a heatmap with the overlapping genes (Supplemental Figure 3A). Results indicate the majority of the genes upregulated in VESCs also showed high expression by the AdvSca1-MA cells. Similar to above, qPCR was used to validate selective overexpression of a subset of the identified genes from this heatmap in AdvSca1-MA cells (Supplemental Figure 3, B and D). In addition, we compared the gene signature specifically within cluster 3 (Figure 1) to the published microarray data from this study. The heatmap in Supplemental Figure 3C shows 21 genes associated with VESCs highly and selectively upregulated in cluster 3; additional genes from Supplemental Figure 5, A and B, found to be upregulated in AdvSca1-MA cells were identified in other regions of the heatmap from Figure 1 that also demonstrated selective expression of genes by AdvSca1-MA cells (Supplemental Figure 3E). These data suggest that AdvSca1-MA cells at least in part represent resident VESCs, but this population likely represents a heterogenous progenitor cell population.

### AdvSca1-SM cells contribute to pathological adventitial remodeling and fibrosis.

**Figure 3:**
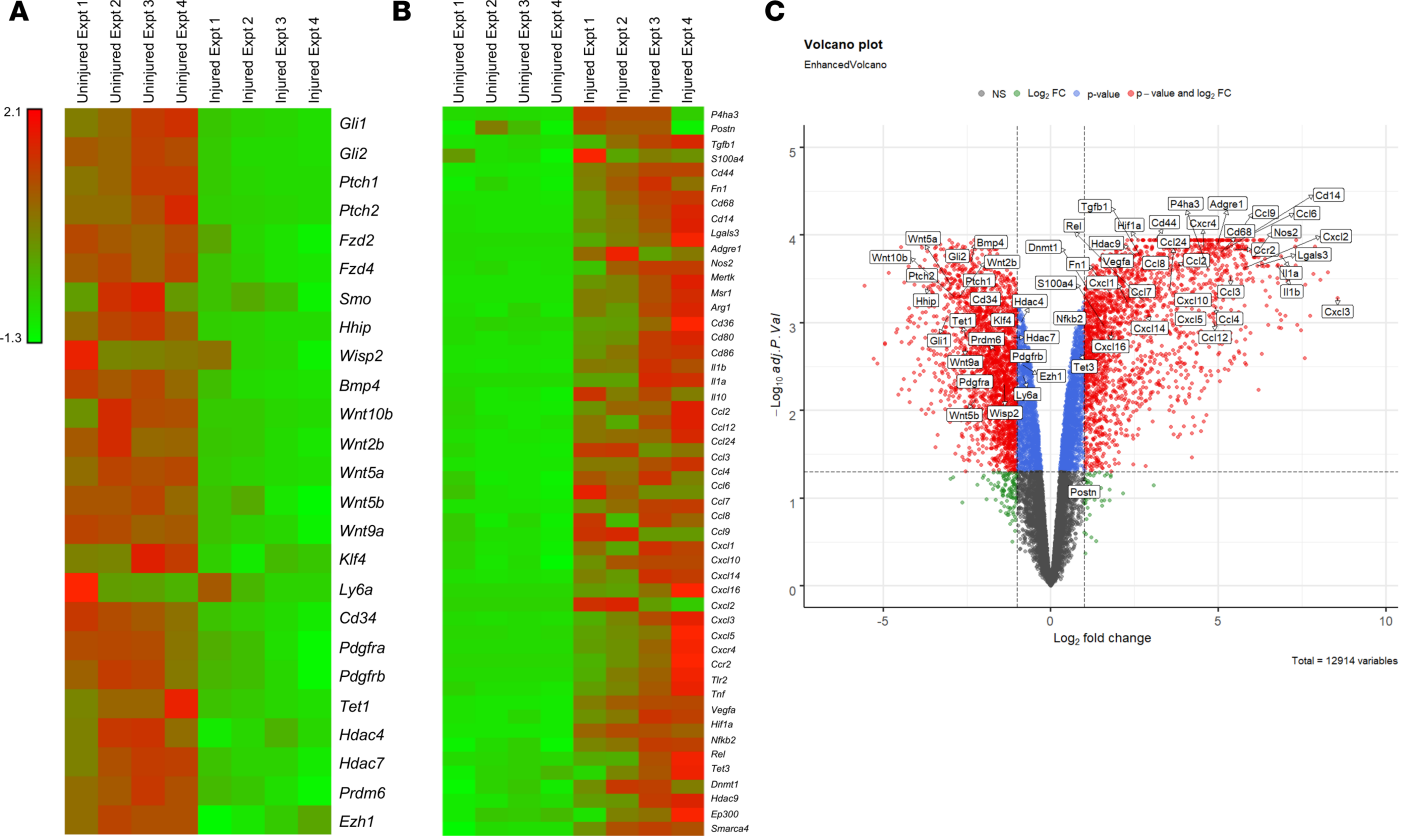
AdvSca1-SM cells downregulate stemness-associated genes and acquire an inflammatory, profibrotic gene signature in response to vascular injury. Two-month-old SMC reporter mice were subjected to carotid artery ligation injury, and injured left and uninjured right arteries were harvested 3 days postinjury for RNA-Seq analysis. AdvSca1-SM cells were recovered from uninjured and injured vessels from 4 separate isolations of cells, with pooled arteries from 12–15 mice contributing to each isolation, and RNA was extracted for RNA-Seq analysis. (**A**) Heatmap of differentially expressed genes demonstrating downregulation of stemness-related genes in AdvSca1-SM cells from injured vessels compared with uninjured vessels. (**B**) Heatmap of differentially expressed genes demonstrating upregulation of myofibroblast- and macrophage-related genes and proinflammatory mediators. Red, upregulated genes; green, downregulated genes. (**C**) Volcano plot displaying differentially expressed genes in AdvSca1-SM cells between uninjured and injured vessels. Negative *x* values represent downregulated genes, and positive *x* values represent upregulated genes in injured vessels compared with uninjured vessels. Horizontal dashed line, adj. *P* = 0.05; vertical dashed line, fold change = 2 or 0.5.

Because little is known regarding the physiological fate and function of AdvSca1-SM cells, the remainder of this study focused on this cell population. We previously demonstrated that AdvSca1-SM cells rapidly and preferentially expand in number in response to vascular injury (25). In order to define the fate and function of AdvSca1-SM cells in response to injury, we first examined the gene expression changes in AdvSca1-SM cells from uninjured compared with injured carotid arteries. For these experiments, mice underwent carotid artery ligation injury as previously described (25, 32). Injured left and uninjured right arteries were isolated 3 days following injury, digested to single-cell suspensions, and flow sorted based on SCA1 and endogenous YFP expression to selectively isolate AdvSca1-SM cells, as described above. RNA was extracted from 4 separate isolations of cells, with arteries from 12–15 mice contributing to each isolation, and subjected to RNA-Seq analysis. As shown in Figure 3A, genes associated with hedgehog/WNT signaling (e.g., *Gli1*, *Gli2*, *Ptch1*, *Wnt2b*, and *Wnt5a*), stemness-associated genes (e.g., *Klf4*, *Ly6a*/SCA1, *Cd34*), and several epigenetic regulators (e.g., *Tet1*, *Hdac7*, *Prdm6*) were found downregulated in AdvSca1-SM cells in response to injury. In contrast, genes associated with a myofibroblast phenotype (e.g., *Postn*, *Tgfb1*, *Fn1*) or with a macrophage phenotype (e.g., *Cd68*, *Lgals3*, *Adgre1*), a large number of inflammatory cytokines/chemokines and associated transcription factors (e.g., *Il1b*, *Ccl2*, *Cxcl1*, *Tnf*, *Nfkb2*), and several epigenetic regulators (e.g., *Dnmt1*, *Hdac9*, *Smarca4*) were found upregulated in AdvSca1-SM cells in response to injury (Figure 3B). The volcano plot showed similar changes in down- and upregulated genes (Figure 3C). Top 20 pathways identified through full pathway analyses conducted through Gene Ontology and KEGG databases are shown in Supplemental Tables 2–5. These data strongly support the hypothesis that AdvSca1-SM cells are activated early in response to injury, downregulate a progenitor-like phenotype, and adopt myofibroblast and/or macrophage phenotypes to promote the proinflammatory, profibrotic environment contributing to pathological vascular remodeling.

In order to test this hypothesis, we generated a specific AdvSca1-SM cell fate-mapping system. Because the original system used to identify AdvSca1-SM cells was based on an SMC-driven reporter, this precluded the ability to selectively track AdvSca1-SM cells in response to vascular injury as both mature SMCs and AdvSca1-SM cells are labeled with reporter. We took advantage of the high and selective expression of *Gli1* by AdvSca1-SM cells (Figure 2 and Supplemental Figure 2) and bred tamoxifen-inducible *Gli1*-Cre^ERT^ transgenic mice to ROSA26-LSL-YFP reporter mice. Following tamoxifen treatment, YFP was expressed exclusively in adventitial SCA1^+^ cells, but not all adventitial SCA1^+^ cells expressed YFP (i.e., SCA1^+^ AdvSca1-MA cells did not express reporter; Supplemental Figure 4A), supporting the use of this system to selectively track AdvSca1-SM cells. To verify SMC origin, RNA-Seq profiling was used to compare gene signatures between SMC reporter AdvSca1-SM cells and *Gli1*-Cre^ERT^-YFP reporter mice. Compared to mature SMCs and non–SMC-derived AdvSca1-MA cells, SMC reporter-derived AdvSca1-SM expressed a similar gene signature as *Gli1*-Cre^ERT^-YFP reporter–derived AdvSca1-SM cells (Supplemental Figure 4B). The reproducibility of the biological replicates for all the conditions examined and the similarity between SMC reporter AdvSca1-SM cells and *Gli1* reporter AdvSca1-SM cells was assessed by principal component analysis and multidimensional scaling plots (Supplemental Figure 4C), thereby supporting the use of *Gli1*-Cre^ERT^-YFP reporter mice as a selective AdvSca1-SM cell fate-mapping system.

**Figure 4:**
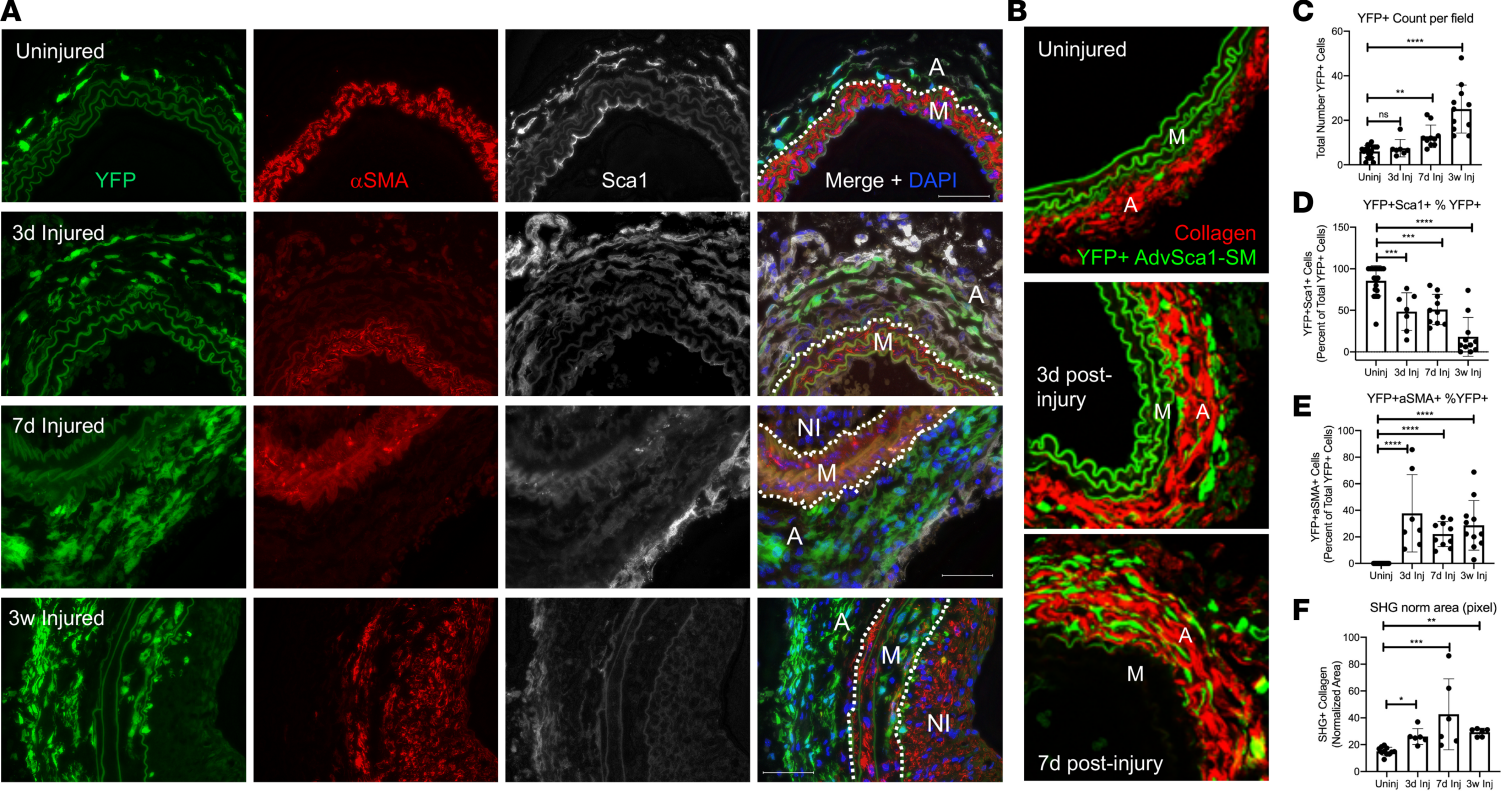
AdvSca1-SM cells contribute to injury-induced adventitial remodeling. *Gli1*-Cre^ERT^-YFP mice were subjected to carotid arterial injury, and uninjured right and injured left carotids were harvested at 3 days, 7 days, and 3 weeks postinjury; fixed; and embedded in OCT. (**A**) Arterial sections were immunofluorescently stained for YFP (green), αSMA (red), and SCA1 (white). Representative images from *N* = 7 (3 days), *N* = 10 (7 days), and *N* = 11 (3 weeks). Scale bars: 50 μm. M, arterial media; A, arterial adventitia, NI, neointima. Dashed lines indicate the external and internal elastic laminae. Note a time-dependent expansion of AdvSca1-SM cells predominantly in the adventitia, with a concomitant loss of AdvSca1-SM cell SCA1 expression, but also migration into the arterial media. (**B**) Uninjured and 3- and 7-day postinjury carotid artery sections were immunofluorescently stained for YFP (green) to identify AdvSca1-SM cells. Sections were imaged for coexpression of YFP and label-free SHG for collagen deposition (red). Elastin autofluorescence is also observed on the green channel. Representative 40× images from *N* = 6 per condition. M, arterial media; A, arterial adventitia. (**C**–**F**) Quantification of stained images from **A** and **B**. (**C**) Total YFP^+^ cells. (**D**) Percentage of YFP^+^SCA1^+^ cells per total YFP^+^ cells. (**E**) Percentage of YFP^+^αSMA^+^ cells per total YFP^+^ cells. (**F**) Normalized SHG+ area (pixel); SHG signal was normalized the outer medial circumference. Data represent mean ± SEM; (**C**, **E**, and **F**) Kruskal-Wallis test followed by Dunn’s multiple-comparison test; (**D**) 1-way ANOVA with Bonferroni’s post hoc test; *P < 0.05; **P < 0.01; ***P < 0.001; ****P < 0.0001.

To determine the contribution of AdvSca1-SM cells to vascular remodeling in response to injury, *Gli1*-Cre^ERT^-YFP mice were treated with tamoxifen, as described in Methods, to permanently label AdvSca1-SM cells in order to selectively track these cells and their progeny in response to injury (15, 25). Tamoxifen-treated *Gli1*-Cre^ERT^-YFP lineage mice were then subjected to carotid artery ligation injury, and uninjured right and injured left carotid arteries were harvested at 3 days, 7 days, and 3 weeks postinjury. As expected, AdvSca1-SM cells in uninjured vessels expressed high levels of SCA1 and undetectable αSMA (Figure 4A). Consistent with our previous findings at 3 days postinjury, AdvSca1-SM cells expanded in number over time in response to injury (Figure 4A). Expansion was associated with increased AdvSca1-SM cell proliferation (Supplemental Figure 5A). Interestingly and quite unexpectedly, AdvSca1-SM cells predominantly accumulated in the arterial adventitia (Figure 4A). While some AdvSca1-SM cells migrated into the media and expressed αSMA, suggesting differentiation into reparative SMCs, very few AdvSca1-SM cells contributed to neointima formation (Figure 4A). Injury-mediated expansion of AdvSca1-SM cells was associated with increased perivascular collagen deposition, as measured by label-free second harmonic generation (SHG) imaging for fibrillar collagen (Figure 4B) and Masson’s trichrome stain (Supplemental Figure 5B). In addition, compared with uninjured control arteries and consistent with our RNA-Seq data (Figure 3), following injury AdvSca1-SM cells gradually lost expression of SCA1, suggesting activation and differentiation of these cells into another cell fate. Importantly, retention of YFP expression by AdvSca1-SM cells permitted faithful tracking and quantification in response to injury even though these cells lost all characteristics of AdvSca1-SM cells (e.g., loss of SCA1 expression). Data were quantified and shown in Figure 4, C–F. To address the relevance and translatability of our findings, we repeated these lineage studies in a separate model of vascular fibrosis, hypertension induced by angiotensin II (Ang-II) infusion. Similar to our results after carotid ligation, AdvSca1-SM cells expanded and accumulated in the adventitia embedded in extensive ECM (Supplemental Figure 6A). Unlike carotid ligation, in which there is damage to the external elastic lamina, Ang-II treatment did not promote movement of AdvSca1-SM cells into the vascular media likely due to Ang-II treatment not inducing as severe damage to the vessel wall.

**Figure 5:**
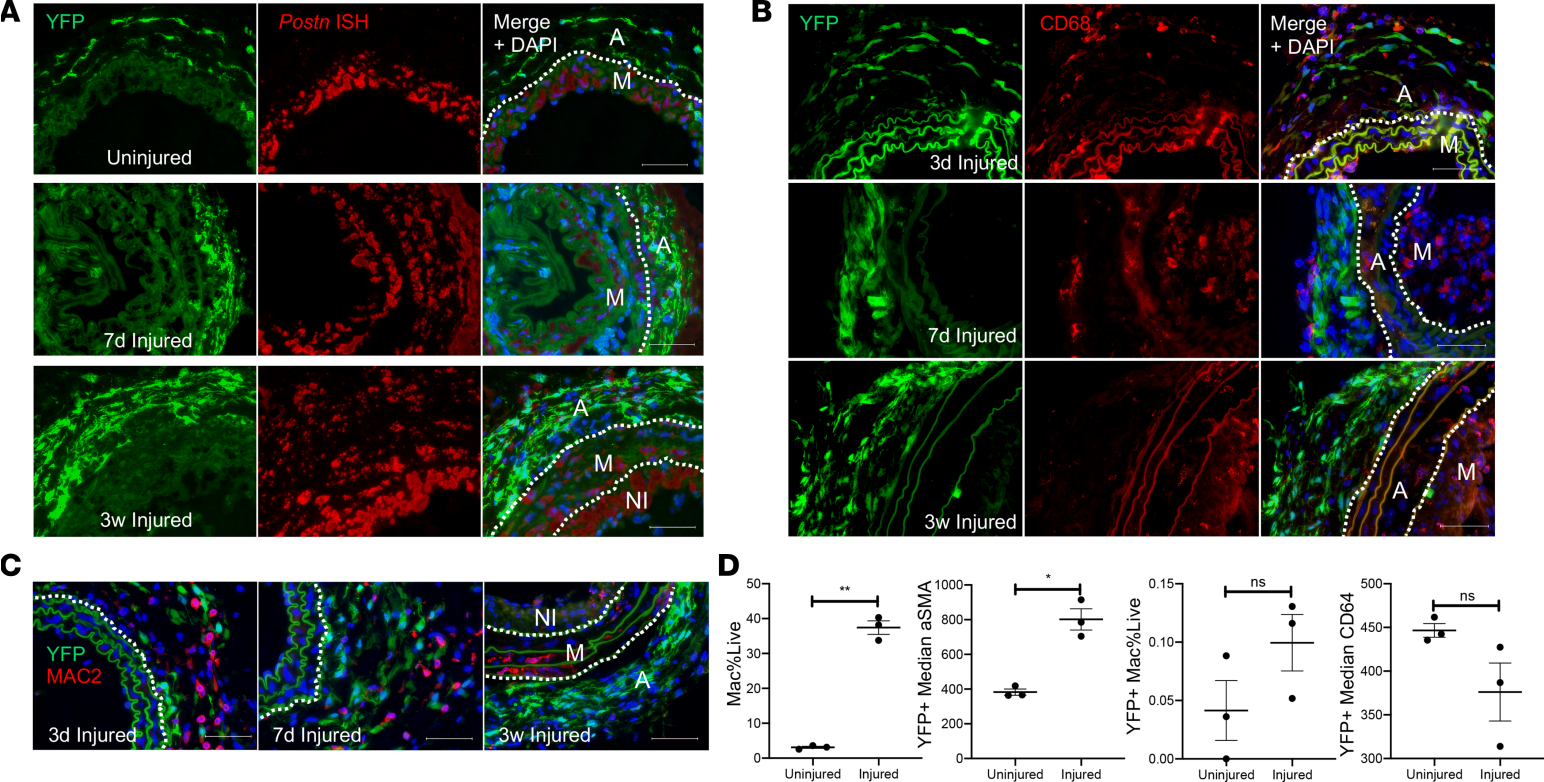
AdvSca1-SM cells adopt a myofibroblast, and only rarely macrophage, phenotype in response to vascular injury. *Gli1*-Cre^ERT^-YFP mice were subjected to carotid arterial injury, and uninjured right and injured left carotids were harvested at 3 days, 7 days, and 3 weeks postinjury; fixed; and embedded in OCT. (**A**) Arterial sections were immunofluorescently stained for YFP (green), and in situ hybridization was used to detect periostin transcripts (red). Representative images from *N* = 3 at each time point. Note strong induction of periostin in AdvSca1-SM cells in response to injury. (**B** and **C**) Arterial sections were immunofluorescently stained for YFP (green) and CD68 (**B**; red) or MAC2 (**C**; red). Representative images from *N* = 3 per time point. Note that AdvSca1-SM cells do not coexpress either macrophage marker. Scale bars for panels **A**–**C**: 50 μm. M, arterial media; A, arterial adventitia; NI, neointima. Dashed lines indicate the external and internal elastic laminae. (**D**) Single-cell suspensions were isolated from uninjured and 7-day postinjured carotid arteries; cells were stained for SCA1, YFP, CD11c, MHCII, LY6G, CD11b, CD64, αSMA, and Aqua VI and analyzed by flow cytometry. Total macrophages (left graph) and YFP^+^ AdvSca1-SM cell-derived macrophages (middle right) were quantified as percentage of all live cells. Median expression of αSMA (middle left) and CD64 (right) by the YFP^+^ cell population was expressed as absolute value. Each point represents an individual carotid artery; *N* = 3. Data represent mean ± SEM; paired Student’s *t* tests (2 tailed); *P < 0.05; **P < 0.01.

Our RNA-Seq data suggest that, in response to injury, AdvSca1-SM cells downregulated a progenitor cell phenotype and upregulated genes associated with a myofibroblast and macrophage phenotype (Figure 3). To test whether AdvSca1-SM cells contribute to myofibroblast or macrophage accumulation in response to injury, uninjured and injured carotid arteries were analyzed for expression of cell-specific markers. Periostin, a secreted matricellular protein that was found upregulated in AdvSca1-SM cells in response to injury, has been identified as a selective marker for activated myofibroblasts (33). In situ hybridization was used to detect periostin transcripts. Compared with uninjured vessels in which there was undetectable periostin in AdvSca1-SM cells, vascular injury promoted a strong induction of periostin that colocalized with AdvSca1-SM cells and collagen deposition (Figure 4B and Supplemental Figure 5B), supporting differentiation into myofibroblasts (Figure 5A and Supplemental Figure 5C). Surprisingly as periostin is believed to be expressed in adult tissues only after injury, periostin mRNA expression was detected in medial SMCs in uninjured vessels but also was detected at high levels in neointimal SMCs following injury, consistent with previous findings (34, 35).

Several reports have indicated that, in the setting of atherosclerosis or cholesterol loading, SMC phenotypic transitions contribute to macrophage-like cells within lesions (22, 23, 36). Since (a) AdvSca1-SM cells originate from mature SMCs, (b) our previous data supported AdvSca1-SM cell differentiation to macrophages in an in vivo Matrigel plug assay, and (c) RNA-Seq data suggested induction of macrophage-specific markers (Figure 3), we hypothesized that activation of AdvSca1-SM cells would contribute to injury-induced inflammation through differentiation into macrophage-like cells. Surprisingly, while vascular injury promoted an inflammatory environment as measured by increased numbers of CD68^+^ and MAC2^+^ macrophages in all vessel layers, we detected only rare YFP^+^ AdvSca1-SM cell–derived macrophages (Figure 5, B and C). Rather, accumulated macrophages, especially in the adventitia, appeared to cluster in close association with AdvSca1-SM cells. While AdvSca1-SM cell differentiation did not significantly contribute to macrophages, these cells expressed high levels of the macrophage chemoattractant, *Ccl2*/MCP-1, consistent with the RNA-Seq data (Supplemental Figure 7, A and B). We used flow cytometry to verify these data. Injured left and uninjured right arteries were isolated 7 days following injury, digested to single-cell suspensions, and labeled with antibodies as described in Methods (Supplemental Figure 8A). As anticipated, we detected an overall increase in macrophage accumulation (Figure 5D, left). Consistent with the imaging data (Figure 4), we detected an increase in αSMA expression by YFP^+^ cells, supporting differentiation of AdvSca1-SM cells into SMCs or myofibroblasts (not only an SMC marker, αSMA is often used to define myofibroblasts) (Figure 5D, center left), but very few YFP^+^ macrophages (Figure 5D, center right and right). Similar to carotid ligation, we observed increased accumulation of CD68^+^ macrophages in response to Ang-II treatment but only rare AdvSca1-SM cell differentiation to macrophages (Supplemental Figure 6B). Collectively, these data highly support the conclusion that, at least in these carotid injury and Ang-II infusion vascular injury models, AdvSca1-SM cells contributed to myofibroblast accumulation and vascular fibrosis but only the rare AdvSca1-SM cell-derived macrophage. However, production of chemokines/cytokines by AdvSca1-SM cells likely promotes macrophage-driven inflammation.

### AdvSca1-SM cell–specific deletion of KLF4 promotes spontaneous adventitial remodeling.

**Figure 6:**
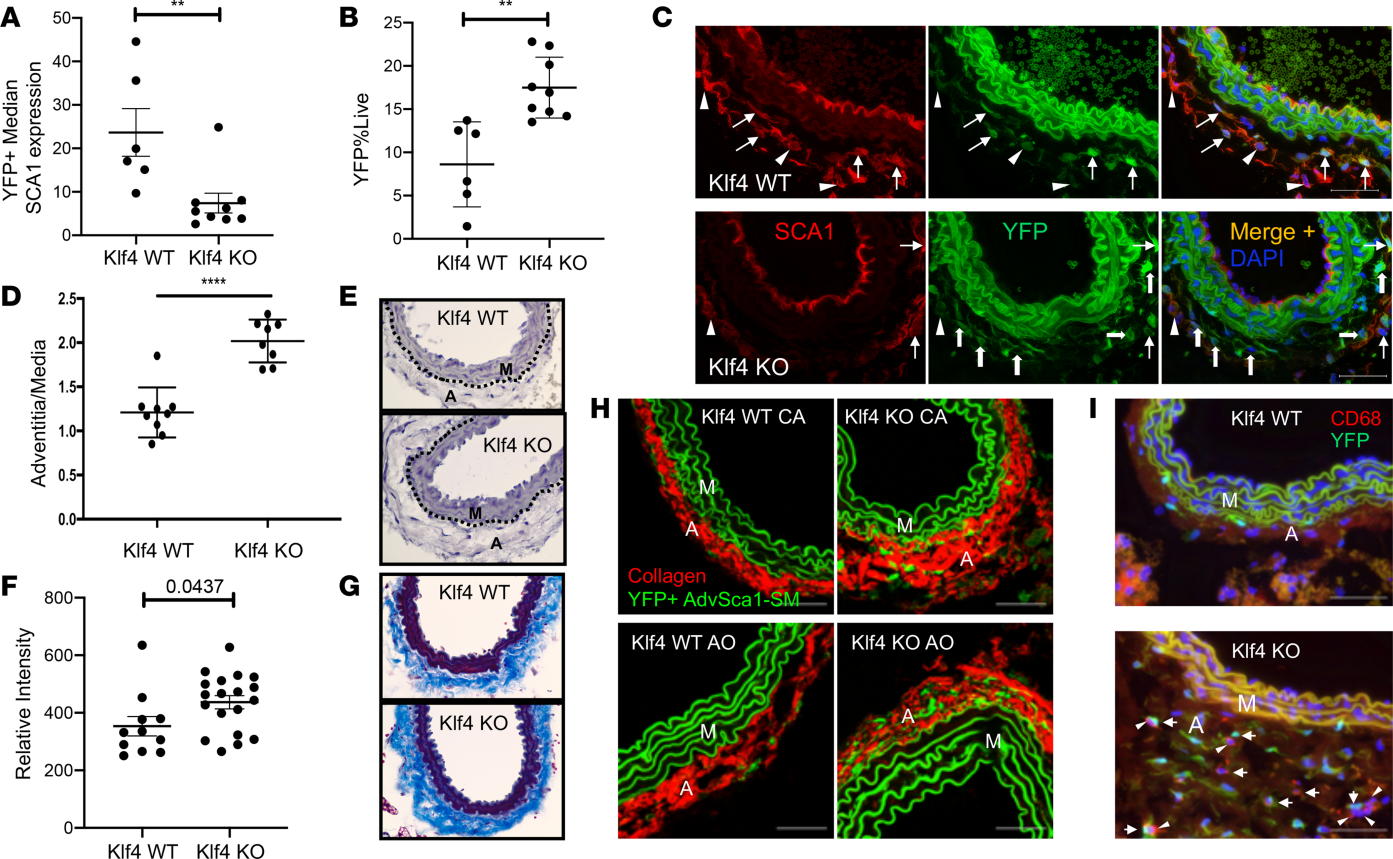
AdvSca1-SM cell–specific deletion of KLF4 promotes spontaneous adventitial remodeling. WT and KLF4-KO *Gli1*Cre^ERT^-YFP mice were injected with tamoxifen as described in Methods. Arterial tissues were harvested 4 weeks after the final tamoxifen injection. (**A** and **B**) Single-cell suspensions were isolated from carotid artery + aortic arch, stained for SCA1, and analyzed by flow cytometry for quantification of SCA1 expression in YFP^+^ AdvSca1-SM cells (**A**) and for quantification of total YFP^+^ AdvSca1-SM–derived cells (**B**). Each point represents a single mouse; *N* = 6 WT and *N* = 9 KO. (**C**) Carotid artery sections were immunofluorescently stained for SCA1 (red) and YFP (green). Arrows show YFP^+^SCA1^+^ AdvSca1-SM cells; block arrows show YFP^+^SCA1^–^ AdvSca1-SM cell-derived cells; arrowheads show YFP^–^SCA1^+^ AdvSca1-MA cells. (**D** and **E**) Carotid artery sections were stained with hematoxylin, and adventitia-to-media ratio was measured using ImageJ 1.47v (NIH) (**D**). Each point represents a single artery. *N* = 9 WT; *N* = 8 KO. Representative image shown in **E**. (**F** and **G**) Carotid artery sections were stained with Masson’s trichrome stain, and the intensity of collagen expression (blue; **G**) was quantified with ImageJ 1.47v and normalized to outer medial circumference (**F**). Each point represents a single artery. *N* = 11 WT; *N* = 19 KO. Representative image shown in **G**. Original magnification, ×40. (**H**) CA and aortic (AO) sections from WT or AdvSca1-SM cell–specific KLF4-KO mice were immunofluorescently stained for YFP (green) to identify AdvSca1-SM cells. Sections were imaged for coexpression of YFP and label-free SHG for collagen deposition (red). Elastin autofluorescence is also observed on the green channel. (**I**) Arterial sections were immunofluorescently stained for YFP (green) and CD68 (red). Representative images from *N* = 3 per time point. Arrows show YFP^+^CD68^–^ AdvSca1-SM cells; arrowheads show YFP^–^CD68^+^ macrophages. Note that AdvSca1-SM cells do not coexpress CD68. M, arterial media; A, arterial adventitia. Scale bars: 50 μm. (**A**, **B**, **D**, and **F**) Data represent mean ± SEM; unpaired Student’s *t* tests (2 tailed); **P < 0.01; ****P < 0.0001.

KLF4, a Kruppel-like transcriptional regulator, is not expressed in differentiated SMCs, but its induction contributes to SMC phenotypic switching (37). KLF4 is 1 of 4 factors necessary for in vitro induced pluripotent stem cell (iPSC) generation, underscoring its important role in the maintenance of a progenitor cell phenotype (38). We previously demonstrated that induction of KLF4 in mature SMCs promotes SMC reprogramming into AdvSca1-SM cells (25). To determine its role in AdvSca1-SM cell phenotype maintenance and function, *Gli1*-Cre^ERT^-YFP lineage mice were bred to *Klf4*-floxed mice to generate AdvSca1-SM cell–specific KLF4-KO mice (*Gli1*-Cre^ERT^-YFP *Klf4*^fl/fl^). Two-month-old *Gli1*-Cre^ERT^-YFP WT and *Gli1*-Cre^ERT^-YFP *Klf4*^fl/fl^ KO mice were injected with tamoxifen to indelibly label AdvSca1-SM cells and to deplete KLF4 selectively in AdvSca1-SM cells of *Gli1*-Cre^ERT^-YFP *Klf4*^fl/fl^ KO mice. Arteries were harvested 4 weeks after the final tamoxifen injection, and flow cytometry was used to quantify SCA1 expression in YFP^+^ AdvSca1-SM cells and to quantify total number of YFP^+^ AdvSca1-SM cells. Compared with WT (*Gli1*-Cre^ERT^-YFP *Klf4*^+/+^), SCA1 expression was decreased in YFP^+^ AdvSca1-SM cells from KLF4-KO mice (Figure 6A and Supplemental Figure 8B), suggesting spontaneous downregulation of a progenitor cell phenotype in the absence of KLF4. Interestingly, this was associated with an overall increase in the number of AdvSca1-SM cells in KLF4-KO mice compared with WT mice (Figure 6B and Supplemental Figure 8C). Immunofluorescence imaging was used to verify the flow cytometry data. Compared with WT mice, KLF4-KO mice exhibited reduced numbers of YFP^+^SCA1^+^ and YFP^+^CD34^+^ AdvSca1-SM cells but increased overall adventitial YFP^+^ AdvSca1-SM–derived cells, the majority of which expressed low to undetectable levels of SCA1 and CD34 (Figure 6C and Supplemental Figure 8D). Because selective depletion of KLF4 in AdvSca1-SM cells appeared to promote spontaneous loss of a progenitor phenotype, we examined the morphology of carotid arteries from WT and KLF4-KO mice. Compared with WT mice, KLF4-KO mice exhibited increased adventitia-to-media ratio, indicating spontaneous adventitial remodeling with loss of KLF4 selectively in AdvSca1-SM cells (Figure 6, D and E). Adventitial remodeling was associated with increased perivascular/adventitial collagen deposition, as measured by Masson’s trichrome staining (Figure 6, F and G) and SHG imaging (Figure 6H). AdvSca1-SM cell loss of KLF4 also promoted increased accumulation of CD68^+^ macrophages, but only very rare YFP^+^ AdvSca1-SM cell-derived macrophages were observed (Figure 6I). Taken together, these results demonstrate that KLF4 is essential for the maintenance of AdvSca1-SM progenitor cell phenotype and loss of KLF4, either genetically or in response to injury (Figure 3), promotes spontaneous myofibroblast differentiation that contributes to vascular remodeling and fibrosis.

## Discussion

**Figure 7:**
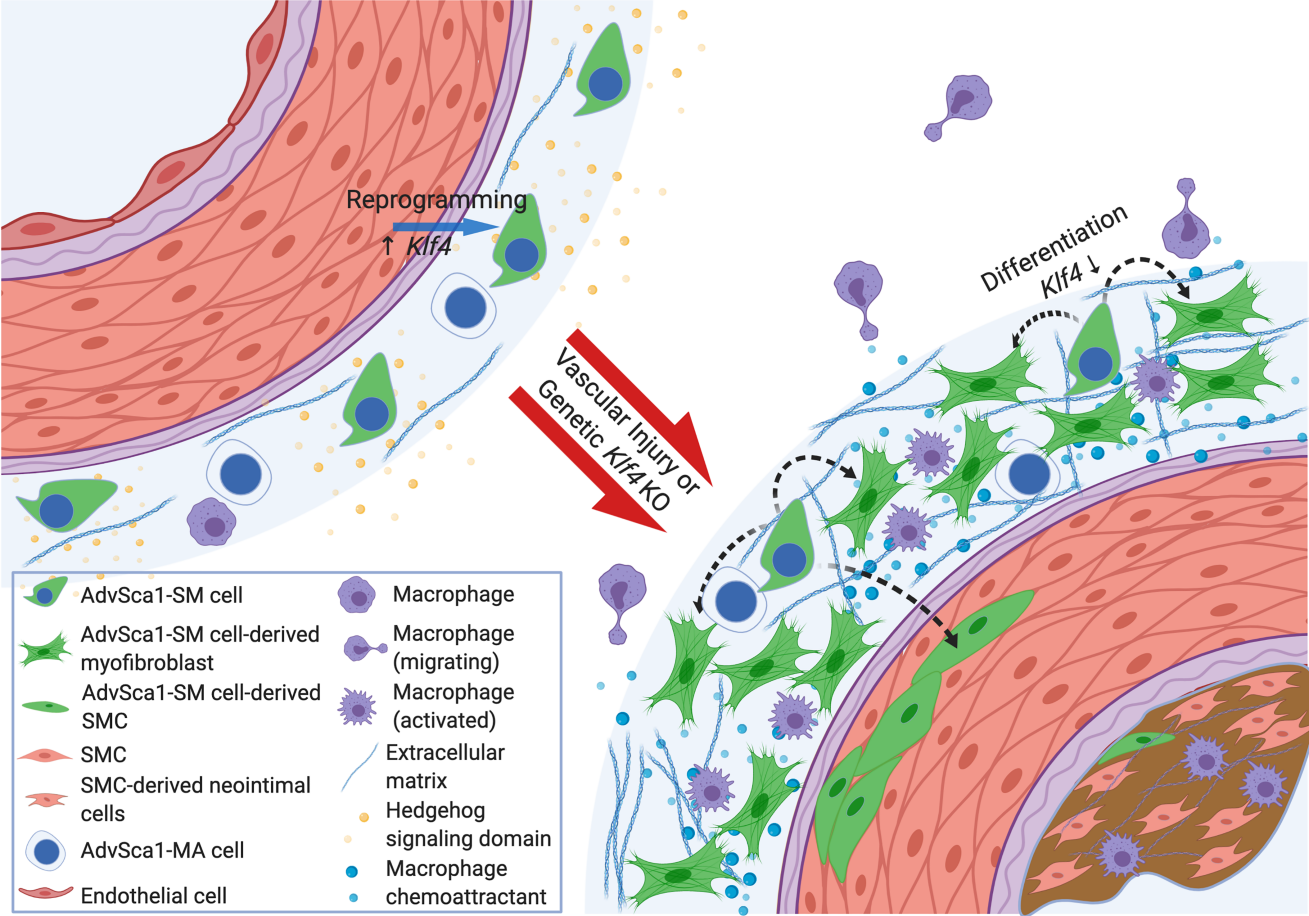
Central role for Klf4-dependent AdvSca1-SM cell contribution to pathological vascular remodeling and fibrosis. AdvSca1-SM cells express a unique gene signature that supports a role for hedgehog/WNT/beta-catenin/KLF4 signaling in regulating SMC-to-AdvSca1-SM cell reprogramming and AdvSca1-SM progenitor cell phenotype and survival. Injury-mediated and/or genetic downregulation of KLF4 disrupts this local progenitor cell niche and promotes activation of AdvSca1-SM cells, as assessed by downregulation of a stemness phenotype. AdvSca1-SM cell activation promotes upregulation of a profibrotic gene signature, thereby facilitating differentiation toward pathological myofibroblasts that contribute to pathological vascular remodeling and fibrosis (created with BioRender.com).

An accurate contribution of resident adventitial progenitor cells (AdvSca1 cells) vis-à-vis other cells residing in the arterial adventitia to vascular disease progression remains poorly understood largely because of the lack of faithful lineage tracking systems. In addition, the discovery that a selective population of AdvSca1 cells is derived from mature SMCs through physiological reprogramming (AdvSca1-SM cells) opens up the possibility that AdvSca1-SM cells could be manipulated in vivo to promote differentiation into reparative SMCs while blocking their differentiation into alternative proremodeling cell types (e.g., profibrotic myofibroblasts, proinflammatory macrophages). Therefore, defining the mechanisms underlying AdvSca1-SM cell phenotype and their contribution to vascular disease will likely uncover novel therapeutic targets. Here, we demonstrate that AdvSca1-SM cells express a unique gene signature that supports a role for hedgehog/WNT/beta-catenin/KLF4 signaling in regulating SMC-to-AdvSca1-SM cell reprogramming and AdvSca1-SM progenitor cell phenotype and survival. In addition, selective expression of multiple ECM and ECM-remodeling components by AdvSca1-SM cells supports their contribution to a unique ECM milieu comprising the local AdvSca1-SM cell progenitor niche. We propose that disruption of this local progenitor cell niche in the setting of vascular injury promotes activation of AdvSca1-SM cells, as assessed by downregulation of a stemness phenotype. In addition, AdvSca1-SM cell activation results in upregulation of profibrotic, proinflammatory gene signatures, thereby facilitating differentiation toward mature cell types that contribute to pathological vascular remodeling (Figure 7).

Over the past decade, the adult vascular adventitia has been identified as being home to multiple resident progenitor cell types. An adventitial “vasculogenic zone” niche was described in adult human arteries and was shown to harbor progenitor cells of multiple phenotypes (8, 10). Several groups, including ours, demonstrated the multipotent differentiation potential of adventitial SCA1^+^ cells into several cell types, including SMCs, macrophages, pericytes, adipocytes, and osteoblasts (7, 9, 12, 39). A subpopulation of SCA1^+^ resident adventitial progenitor cells coexpressing the hematopoietic marker, CD45, was shown to exhibit monocyte progenitor properties and to contribute to low-level hematopoiesis (11, 17). Our previous work showed that adventitial SCA1^+^ cells are clustered in a zone of sonic hedgehog (SHH) signaling and depletion of *Shh* results in much reduced numbers of aortic adventitial SCA1^+^ cells, supporting a role for this pathway in their maintenance and survival (9). Our findings here that AdvSca1-SM cells exhibit constitutive hedgehog signaling as assessed by selective expression of hedgehog-responsive genes such as *Gli1* and *Gli*2 and *Ptch1* and *Ptch2* are fully consistent with these previous findings. The majority of studies focused on the role of adventitial SCA1^+^ cells in vascular disease support the concept that these cells facilitate disease development and progression. However, most studies that have focused on these cells examined heterogenous populations of AdvSca1 cells rather than defining distinct functions of phenotypically unique subpopulations. In this context, therefore, a major goal would be to harness the potential of specific adventitial SCA1^+^ progenitor cells to differentiate into specific cell types in situ to promote tissue regeneration and block pathological vascular fibrosis and stiffening. A major challenge in the field has been a lack of understanding of the progenitor cell niche and specific signaling pathways and mechanisms regulating distinct populations of adventitial SCA1^+^ progenitor subtypes and their response to disease. The data presented here demonstrate that AdvSca1-SM cells express a unique gene signature involved in regulation of their identity and functionally important in the maintenance of their stemness phenotype. Inhibiting downregulation of these signals in the setting of disease may possibly block their contribution to pathological vascular remodeling. Alternatively, blocking induction of signals found upregulated in the setting of disease could be critical to promote differentiation toward a reparative SMC phenotype and thus contribute to vessel homeostasis and repair rather than pathological vascular remodeling.

The use of a physiologically relevant and reproducible lineage tracing model that selectively tracks a specific subtype of resident vascular progenitor cells is crucial for a full understanding of the physiological and pathophysiological roles of these cells. Guided by our RNA-Seq findings demonstrating AdvSca1-SM cells uniquely expressed high levels of the transcription factor, *Gli1*, we generated an AdvSca1-SM cell fate-mapping system. Compared to the SMC fate-mapping system used to identify AdvSca1-SM cells, *Gli1*-Cre^ERT^–derived AdvSca1-SM cells exhibited an identical gene signature, establishing the use of this system to selectively and physiologically track these cells in response to vascular disease. Similar to others’ and our previous reports (25, 40), using this system, AdvSca1-SM cells quickly and robustly expanded in number in response to injury to contribute to adventitial remodeling. Surprisingly, at least in the setting of carotid artery ligation injury, AdvSca1-SM cells predominantly contributed to adventitial remodeling and fibrosis. These cells adopted a myofibroblast phenotype as characterized by high periostin expression. Despite RNA-Seq data suggesting induction of macrophage-specific markers, such as *Adgre1*/F4/80, *Lgals3*/MAC2, and CD68, in response to injury, we detected only rare YFP^+^CD68^+^/MAC2^+^ cells. However, consistent with postinjury RNA-Seq data, AdvSca1-SM cells contributing to adventitial remodeling expressed high levels of the macrophage chemoattractant, *Ccl2*/MCP1, early in response to injury, suggesting production of inflammatory mediators promotes macrophage recruitment and expansion. These findings are in contrast to our previous data that demonstrated the potential for macrophage differentiation; however, these previous studies were conducted using a somewhat artificial in vivo Matrigel plug assay. Therefore, it is most likely that differentiation potential is contextually driven. In contrast to earlier studies finding AdvSca1 cell contribution to intimal hyperplasia in the setting of severe arterial injury, very few AdvSca1-SM cells contributed to neointimal hyperplasia. However, this finding is fully consistent with our previous work using our SMC lineage-tracking system that demonstrated mature SMCs are the major contributors to intimal hyperplasia (15, 25). Differences in injury models likely explain the opposing findings as we examined a less severe form of vascular injury compared with previous reports. Alternatively, compared with our system, which specifically tracked AdvSca1-SM cells, previous studies examined the entire heterogenous population of AdvSca1 cells. Finally, fairly significant numbers of αSMA^+^ AdvSca1-SM cells were detected in the arterial media. This finding supports a role for AdvSca1-SM cell migration into the media and differentiation toward mature SMCs as a mechanism to facilitate vessel repair. It should be pointed out that we cannot rule out the possibility of a rare medial SMC or medial progenitor cell expressing *Gli1* that expands in response to injury to contribute to medial repair. In fact, examination of published single-cell RNA-Seq data from mouse arteries indicated this possibility (41). As discussed above, targeting mechanisms promoting the SMC phenotype while blocking profibrotic signaling driving a myofibroblast phenotype could lead to novel therapeutics that address pathological vascular adventitial remodeling and fibrosis.

It is important to point out that, while careful lineage tracing was performed for these studies, because of limitations of our study, the identity and lineage of AdvSca1-SM cells likely have not been fully elucidated. In addition, we acknowledge that *Gli1* is not lineage specific and that many other cell types have been shown to express *Gli1* in the context of hedgehog signaling. Our original report identifying AdvSca1-SM cells used a *Myh11*-Cre^ERT^ reporter system in which *Myh11* is a highly selective marker for mature SMCs. We demonstrated very selective reporter expression in SMCs and no expression in other cell types, including bone marrow cells (15). In addition, previous studies using bone marrow transplant approaches demonstrated that bone marrow–derived cells do not contribute to the AdvSca1 cell population (7, 11). However, given recent sensitive technology with single-cell RNA-Seq approaches, at this point we cannot rule out the possibility that other nonvascular, non–AdvSca1-SM *Gli1*^+^ cells contribute to the observed vascular remodeling, in particular bone marrow–derived cells that also express *Myh11*.

Differentiation of AdvSca1-SM cells into myofibroblasts in the setting of vascular injury contributes to marked pathological perivascular fibrosis and vessel stiffening. In contrast, it is possible that in the setting of atherosclerosis, AdvSca1-SM–derived myofibroblasts might exert protective effects through the maintenance of the fibrous cap and reduced destabilization of advanced lesions. In contrast to previous work (22, 23, 36), a recent intriguing paper employing a similar *Myh11-*Cre^ERT^ SMC fate-mapping system and single-cell RNA-Seq approaches in the setting of atherosclerosis reported that mature SMCs transition to “fibromyocytes” rather than macrophages to contribute to fibrous cap formation and lesion stability (42). Moving forward, an interesting question will be to determine if, in this setting, mature SMCs undergo a transient reprogramming process to AdvSca1-SM cells, which then transition into protective fibromyocyte-like cells. Pertinent to this, comparing our RNA-Seq data from AdvSca1-SM cells to the single-cell RNA-Seq data presented in this manuscript, we found a similar gene signature between AdvSca1-SM cells and the previously identified fibromyocytes (our unpublished data).

KLF4 is a member of the Kruppel-like family of transcriptional regulators. KLF4 contains both activation and repression domains that function by recruiting transcriptional coactivators or corepressors to regulate gene transcription (43). Importantly, KLF4 is well known for its role in reprogramming somatic cells into iPSCs (38). Our previous work demonstrated that induction of KLF4 in mature SMCs is essential for reprogramming SMCs into AdvSca1-SM cells (25). SMC-specific depletion of KLF4 resulted in a selective reduction of AdvSca1-SM cells but not AdvSca1-MA cells. Others have shown that SMC-specific depletion of KLF4 attenuates experimental atherosclerosis lesion formation and abdominal aortic aneurysm formation (23, 44). Collectively these data and previous studies support an important role for KLF4 in regulating SMC phenotypic transitions. While well known for regulating embryonic stem cell and iPSC self-renewal, little is known regarding its role in maintenance of somatic tissue-specific progenitor cell phenotype and, in particular, AdvSca1-SM cells. In the current study we report that AdvSca1-SM cell expression of KLF4 is essential for maintenance of the progenitor cell phenotype. Surprisingly, rather than decreased AdvSca1-SM cell survival, AdvSca1-SM cell–specific KLF4 depletion facilitated spontaneous expansion of AdvSca1-SM cells and loss of a stemness phenotype as characterized by loss of SCA1 and CD34 expression in YFP^+^ AdvSca1-SM-derived cells. This was associated with spontaneous adventitial remodeling, perivascular fibrosis, and macrophage accumulation, consistent with our findings that AdvSca1-SM cells adopt a profibrotic myofibroblast-like phenotype in response to vascular injury to contribute to pathological adventitial remodeling. These results suggest that targeting signals responsible for downregulating KLF4 could be potential therapeutic approaches to maintain AdvSca1-SM progenitor cell phenotype and block contribution to pathological vascular remodeling.

In addition to SMC-derived AdvSca1-SM cells, we previously described an additional subpopulation of adventitial SCA1^+^ progenitor cells that we termed AdvSca1-MA cells. While this population of progenitor cells likely consists of additional unique subpopulations, RNA-Seq profiling identified AdvSca1-MA cells as representing resident endothelial cell progenitors, which is consistent with the top pathways being related to Notch signaling, NO pathway, cell-cell junction organization, and VEGF signaling. Compared with AdvSca1-SM cells, AdvSca1-MA cells expressed classic endothelial progenitor markers (e.g., *Mcam*/CD146, *Pecam*/CD31, *Cdh5*/Ve-cadherin, *Kdr*/FLK1, *Flt1*, *Fli1*, *Vwf*, *Prom1*, *Tek*/TIE2, *Bmx*) as well as the cell surface marker *Bst1*/CD157, previously identified as a marker of tissue-resident VESCs that were shown to exist in major vessels of several organs (31). These cells, termed tissue-resident VESCs, were shown to expand in number to contribute to regeneration of vascular structures. Given the high degree of similarity in transcriptomes, we propose that AdvSca1-MA cells at least in part represent resident VESCs. However, the AdvSca1-MA cell subpopulation likely consists of additional subtypes of AdvSca1 progenitor cells, as our previous report identified a CD45^+^SCA1^+^ subtype that represented approximately one-third of all AdvSca1-MA cells. We propose that this subtype is likely the previously described myeloid progenitor cell population (11). Future studies employing single-cell RNA-Seq approaches will be useful to fully characterize the heterogeneity of this subtype of AdvSca1 cells.

In summary, using RNA-Seq and AdvSca1-SM cell–specific fate-mapping, our findings confirm that induction of hedgehog/KLF4 signaling underlies SMC reprogramming to SMC-derived AdvSca1-SM cells. These cells represent a defined subtype of resident heterogenous populations of vascular progenitor cells identified by us and others. Using a physiologically relevant vascular disease model, while some AdvSca1-SM cells contributed to vessel repair, the predominant pathological function involved contribution to vascular fibrosis with little contribution to neointima formation. This process involved downregulation of *Klf4* activity and induction of a profibrotic myofibroblast phenotype. Therefore, targeting critical mechanisms promoting the reparative SMC phenotype while blocking profibrotic signaling pathways could lead to novel therapeutics that target pathological vascular adventitial remodeling and fibrosis.

## Methods

### Mice and vascular injury.

*Klf4*-floxed mice were obtained from Klaus H. Kaestner (University of Pennsylvania, Philadelphia, Pennsylvania, USA). SM22α-Cre (*TagIn*-Cre; stock 004746), *Gli1*-Cre^ERT^ (stock 007913), and ROSA26-YFP reporter mice (stock 006148) were obtained from The Jackson Laboratory. All mice were fully backcrossed to a C57BL/6 genetic background prior to studies. SM22α-Cre transgenic mice were bred to ROSA26-YFP to generate SMC-specific YFP-expressing reporter mice (SM22α-Cre-YFP). *Gli1*-Cre^ERT^ transgenic mice and ROSA26-YFP reporter mice were bred to generate tamoxifen-inducible AdvSca1-SM cell–specific YFP-expressing reporter mice (*Gli1*-Cre^ERT^-YFP). *Gli1*-Cre^ERT^-YFP mice were bred to *Klf4*-floxed mice to generate AdvSca1-SM cell–specific *Klf4*-KO mice (*Gli1*-Cre^ERT^-YFP *Klf4*^fl/fl^). Adult *Gli1*-Cre^ERT^-YFP mice received 1 mg IP tamoxifen injections for 12 consecutive days to induce YFP reporter knockin and *Klf4* KO prior to experiments. To induce vascular injury, mice were subjected to total carotid artery ligation injury (25, 32). Mice were anesthetized with isoflurane and left carotid arteries of 2-month-old mice were completely ligated just proximal to the carotid bifurcation. Uninjured and injured arteries were harvested 3 days, 7 days, and 3 weeks following carotid artery ligation injury, perfusion fixed in 4% buffered paraformaldehyde (PFA), and embedded in OCT (Fisher Healthcare catalog 4585) for immunofluorescence staining. In separate studies, arteries were isolated from uninjured and injured carotid arteries of SM22α-Cre-YFP mice, pooled, and digested to single-cell suspensions, with AdvSca1-SM cells flow sorted for RNA-Seq analysis. For *Klf4*-KO studies, *Gli1*-Cre^ERT^-YFP WT and *Gli1*-Cre^ERT^-YFP *Klf4*^fl/fl^ KO mice were injected with tamoxifen and arteries harvested 4 weeks after the last tamoxifen injection. To assess cell proliferation, mice were injected with 5-ethynyl-2′-deoxyuridine (50 mg/kg IP; Carbosynth catalog NE08701EdU) at 5 pm the evening prior to sacrifice and 8 am the morning of sacrifice. Both male and female mice were employed and randomly assigned to experimental groups in all studies. For Ang-II experiments, *Gli1*-Cre^ERT^-YFP mice were subjected to Ang-II/saline treatments after 12 consecutive days of tamoxifen injections (1 mg IP) and 7 days of washout. All surgeries were performed in a clean environment with sterile instruments. Micro-osmotic pumps (ALZET model 1004) were filled with calculated concentrations of Ang-II or saline adjusted to the body weight of each individual mouse to ensure the delivery of Ang-II at the rate of 1 μg/kg/min per manufacturer’s instructions. The pumps were primed in sterile saline at 37°C for 24 hours. The primed pumps were inserted subcutaneously through a small incision at the dorsal neck region of the isoflurane-anesthetized mouse. The incision was closed with stainless steel wound clips (CellPoint Scientific). After 28 days of treatment, the mice were euthanized with isoflurane, and aortic tissues were dissected, fixed in 4% buffered PFA, and embedded in OCT for immunofluorescence staining and SHG imaging.

### Preparation of single-cell suspensions and flow cytometry.

Eight-week-old SM22α-Cre-YFP mice were sacrificed, the vasculature was perfused with heparinized saline (20 U/mL, MilliporeSigma), and descending aortas and combined aortic arch plus left and right carotid arteries were digested to single cells by digestion at 37°C for 1 hour in collagenase buffer (3.2 mg/mL collagenase II, 0.7 mg/mL elastase from Worthington, 0.2 mg/mL soybean trypsin inhibitor from MilliporeSigma in HBSS, pH 7.5) (25). Arteries were harvested under sterile conditions following flushing of the vasculature with sterile heparinized PBS (20 U/mL, MilliporeSigma) and minced prior to digestion. During incubation samples were dispersed by pipetting every 10 minutes. Single-cell suspensions were passed through a 70 μm filter and washed twice with sterile PBS + 0.1% calf serum (CS). For flow sorting, single-cell suspensions were suspended in 100 μL PBS + 0.1% CS and incubated with 3 μL of a rat anti-mouse monoclonal SCA1 antibody (APC conjugated; eBioscience, Thermo Fisher Scientific, catalog 17-5981-82); live cells were sorted based on SCA1-APC and endogenous YFP expression. The gating strategy for sorting is shown in Supplemental Figure 1. Three distinct cell populations were sorted: YFP^+^SCA1^–^ mature SMCs, YFP^+^SCA1^+^ SMC-derived AdvSca1-SM cells, and YFP^–^SCA1^+^ non-SMC AdvSca1-MA cells. Sorting was performed on a MoFlo high-speed cell sorter. For RNA-Seq data shown in Figure 1 and Figure 2 and Supplemental Figures 2, 5, 6, and 7, *N* = 3 independent experiments using pooled arteries from 10–14 mice per experiment for analysis. High-quality RNA was obtained for analysis of *N* = 3 mature SMCs and *N* = 2 AdvSca1-SM and AdvSca1-MA cells. For RNA-Seq data shown in Figure 3, *N* = 4 independent experiments using pooled arteries from uninjured and injured carotid arteries from 15 mice per experiment for analysis. For RNA-Seq analysis shown in Supplemental Figure 7, individual arteries from *N* = 6–9 mice were used for analysis. For FACS analysis, single-cell suspensions were stained with the following antibodies: anti-SCA1 (APC-Cy7 conjugated; BD Bioscience catalog 560654), anti-CD11b (PerCP/Cy5.5 conjugated; BioLegend catalog 101228), anti-CD11c (PE conjugated; BD Bioscience catalog 561044), anti-MHCII (eFluor450 conjugated; Invitrogen, Thermo Fisher Scientific, catalog 48-5321-82), anti-LY6G (PE/Cy7 conjugated; BioLegend catalog 127617), anti-CD64 (PE/Dazzle594 conjugated; BioLegend catalog 139320), anti-αSMA (eFluor660 conjugated; eBioscience, Thermo Fisher Scientific, catalog 50-9760-82), anti-GFP (FITC conjugated; Abcam catalog ab6662), and LIVE/DEAD Fixable Aqua Dead Cell Stain Kit (Aqua VI dye; Molecular Probes, Thermo Fisher Scientific, catalog L34957) to stain for dead cells. Isotype-matched control antibodies were used. Flow cytometry was performed on a Galios cytometer (Becton Dickinson). Data were analyzed using Kaluza Software (Beckman Coulter). The gating strategy for flow cytometry is shown in Supplemental Figure 8A. Briefly, live cells were plotted for endogenous YFP and αSMA expression and Aqua to gate out dead cells and identify and track YFP^+^ AdvSca1-SM cells for expression of macrophage markers. To quantitate SCA1 expression and total YFP^+^ AdvSca1-SM–derived cells, arteries from *Gli1*-Cre^ERT^-YFP WT and *Gli1*-Cre^ERT^-YFP *Klf4*^fl/fl^ KO mice were individually digested to single-cell suspensions, labeled with APC-conjugated anti-SCA1 antibodies, and analyzed for endogenous YFP and SCA1-APC expression.

### RNA extraction and RNA-Seq.

Total RNA was extracted from flow cytometry–sorted cells using QIAshredders and an RNeasy Plus Micro kit (QIAGEN) (25, 45). RNA quality and quantity were analyzed using a NanoDrop and Bioanalyzer. RNA-Seq library preparation and sequencing were conducted at the Genomics and Microarray Core at the University of Colorado Anschutz Medical Campus (46). Libraries were constructed using a SMARTer Stranded Total RNA-Seq kit (Clontech) customized with mouse-specific oligonucleotides for rRNA removal. Directional mRNA-Seq of sorted cell populations was conducted using the Illumina HiSeq 2500 or 4000 system.

### Bioinformatics analysis.

RNA-Seq reads were obtained using Illumina HiSeq analysis pipeline, as previously described (46). Prealignment read quality was performed using FastQC (http://www.bioinformatics.bbsrc.ac.uk/projects/fastqc). Reads were then processed and aligned to the University of California, Santa Cruz, *Mus musculus* reference genome (build mm10) using HISAT2 version 2.1.0 with default settings (47). Cufflinks version 2.2.1 was employed to assemble the transcript from the aligned sequencing data, estimate their abundance, and test for differential expression (48). Differentially expressed genes, identified by the criteria of FPKM > 1 and 2-fold change with a false discovery rate < 0.05 in pairwise comparisons, were selected by a custom python script for hierarchical clustering using Cluster 3.0 (49). Clustering was performed with the complete linkage and Euclidean distance as the metric and visualized with Java Tree View as a heatmap (50). Gene set overrepresentation analysis of genes in clusters of interest was performed using ConsensusPathDB (27) to identify enriched pathways in KEGG and Reactome databases with cutoff values of *P* < 0.01 and minimum of 3 overlapping genes. Additionally, GSEA version 3.0 (28) was performed to confirm enrichment of pathways in select cell populations. For carotid artery injury RNA-Seq experiments, sequencing alignment was performed with HISAT2 (47) (version 2.1.0), and raw gene counts were generated with HTSeq (51) (version 0.10.0) for subsequent analysis. The R package *limma* (52) (version 3.44.3) was used to perform pairwise comparison between the injured and uninjured carotid artery samples from each experiment to generate differentially expressed genes (adj. *P* < 0.05, log fold change > 1 or < –1). The R package EnhancedVolcano (version 1.6.0) was employed to generate the volcano plot. Normalized counts per million data were used to generate the heatmap with Matrix2png (53). Differentially expressed gene lists were subjected to Gene Ontology enrichment analysis using PANTHER classification system (54) and KEGG pathway analysis using DAVID (55, 56). Bubble plots of the analysis results were generated with the R package ggplot2 (version 3.3.2). For the comparison between gene expression profile of cell populations from SM22α-Cre-YFP mice and *Gli1*-Cre^ERT^-YFP mice, cufflink analysis output was used in CummeRbund to generate principal component analysis and multidimensional scaling plots (48). Hierarchical clustering was performed with FPKM data of the differentially expressed genes identified as described above. The accession number for the RNA-Seq data reported in this paper is GSE145569 (NCBI Gene Expression Omnibus database).

### Immunofluorescence, microscopy, in situ hybridization, SHG imaging, and Masson’s trichrome staining and quantification.

For immunofluorescence staining, arteries were fixed in 4% PFA and embedded in OCT for sectioning (25). Tissue sections were permeabilized with MeOH for 10 minutes followed by 0.05% Tween-20 in PBS for 5 minutes, blocked in 3% horse serum, and sequentially incubated with specific primary and secondary antibodies. Antibodies used include monoclonal rat anti-mouse SCA1 (1:100; BD Pharmingen catalog 553333), FITC-conjugated polyclonal goat anti-GFP (1:250; Abcam catalog 6662), Cy3-conjugated monoclonal anti–αSMA (1:2000; MilliporeSigma catalog C6198), monoclonal anti-CD68 (1:50; BioRad catalog MCA1957), and monoclonal anti-LGALS3 (1:100; Life Technologies, Thermo Fisher Scientific, catalog 14-5301). For unconjugated antibodies, antigen/antibody complexes were visualized using Alexa Fluor 488–, Alexa Fluor 568–, or Alexa Fluor 647–coupled secondary antibodies (Invitrogen, Thermo Fisher Scientific, catalog A-11006, A-11077, and A-21247). Slides were mounted with VECTASHIELD medium containing DAPI (VECTASHIELD Antifade Mounting Medium with DAPI; catalog H-1200, VECTOR Laboratories) to detect all cell nuclei and tissues imaged using a laser-scanning confocal microscope (LSM 780 spectral, Carl Zeiss) with a 63× oil immersion objective or a Keyence BZ-X710 all-in-one fluorescence microscope with a 60× oil immersion objective. Images were analyzed using ZEN LE software (LSM 780) or BX-X Analyzer software (Keyence). Negative controls included the use of rat or rabbit IgG (SouthernBiotech catalog 0108-01 and Invitrogen, Thermo Fisher Scientific, catalog 31235). For SCA1-stained tissues, sections were hydrated, blocked in 3% horse serum, incubated overnight with the SCA1 antibody, washed in PBS, and then permeabilized prior to secondary antibody incubation. Click-iT EdU Cell Proliferation Kit for Imaging (Invitrogen, Thermo Fisher Scientific, catalog C10339) was employed to assess cell proliferation. Sections from EdU-injected mice were washed twice with 1 mL of 3% BSA in PBS and then incubated with 0.5 mL of Click-iT reaction cocktail (Invitrogen, Thermo Fisher Scientific, catalog C10339) for 30 minutes at room temperature in the dark. Fluorescence in situ hybridization staining for periostin transcripts was performed using Advanced Cell Diagnostics RNAscope 2.5 HD Red Assay using the standard protocol with the Mm-Postn probe (catalog 418581). Positive control probe used was Mm-Ppib (catalog 313911) and negative control was DapB (catalog 310043). Following completion of the in situ hybridization protocol, sections were then stained for presence of YFP with FITC-conjugated anti-GFP (as above). To analyze collagen deposition, arterial sections were immunofluorescently stained for YFP to identify AdvSca1-SM cells and sections imaged for YFP expression and label-free SHG using a laser-scanning confocal microscope (LSM 780). In addition, sections were stained for Masson’s trichrome stain; staining was performed at the University of Colorado Denver Tissue Biobanking and Histology Shared Resource. The intensity of positive collagen expression was quantified with ImageJ 1.47v and normalized to outer medial circumference. Imaging for Masson’s-stained tissues and H&E-stained tissues was performed on an Olympus BX41 bright-field/phase contrast microscope using a 40× objective, and image analysis was performed using Spot 5.3 software. Quantification of the immunofluorescence image was performed with CellProfiler (version 4.0.4) and visually confirmed to exclude false positives. SHG quantification was performed on 20× images.

### Quantitative real-time PCR.

Total RNA was isolated from flow-isolated cell populations by first digesting in RLT lysis buffer (QIAGEN) (25, 45). Samples were then processed with QIAshredder and RNeasy Plus kits (QIAGEN) to isolate RNA. First strand cDNA was made using the qScript XLT cDNA SuperMix synthesis kit (Quantabio). Sequence-specific primers were designed (Supplemental Table 1). Quantitative real-time PCR was performed as previously described (25, 45) and GAPDH was used for normalization. Data were normalized to YFP^+^ mature SMCs (YFP SMC set to 1 to average among independent experiments).

### Statistics.

Data were expressed as means ± SEM. All experiments reported were carried out with at least 3 biological replicates, including both male and female mice when possible. The *N* values in the study, representing the number of biological replicates, were reported in the corresponding figure legends. We found no differences between males and females; therefore, the data were combined for analysis. Data were analyzed using GraphPad Prism 7 (GraphPad Software, Inc). Shapiro-Wilk test or D’Agostino-Pearson test was performed to determine the normality of the data. Brown-Forsythe test was used to examine the equality of group variances. Unpaired Student’s *t* test was performed for comparing between 2 groups. Paired Student’s *t* test (2 tailed) was employed for the statistical analysis of flow cytometry data comparing injured and uninjured carotid samples. One-way ANOVA with Bonferroni’s post hoc test was used to compare multiple groups. *P* values less than 0.05 were considered statistically significant. For data that failed the normality test or equal variances test, a Kruskal-Wallis test was used to compare the groups followed by Dunn’s multiple-comparison tests.

### Study approval.

Mice were maintained in the Center for Comparative Medicine, and procedures were performed under a protocol approved by the Institutional Animal Care and Use Committee at the University of Colorado Denver.

## Author contributions

SL and MCMWE designed the studies. SL, AJJ, KAS, AMD, and MFM performed experiments. SL performed all bioinformatics associated with this study. KAS assisted with mouse surgeries and tissue harvest. MFM managed mouse colonies associated with this project and served as lab manager for the MCMWE lab. SL, MWM, and MCMWE analyzed and interpreted the experimental data. SL and MCMWE wrote the manuscript. AJJ, KAS, AMD, KSM, RAN, and MWM edited the manuscript.

## Supplementary Material

supplemental data

## Figures and Tables

**Table 1:**
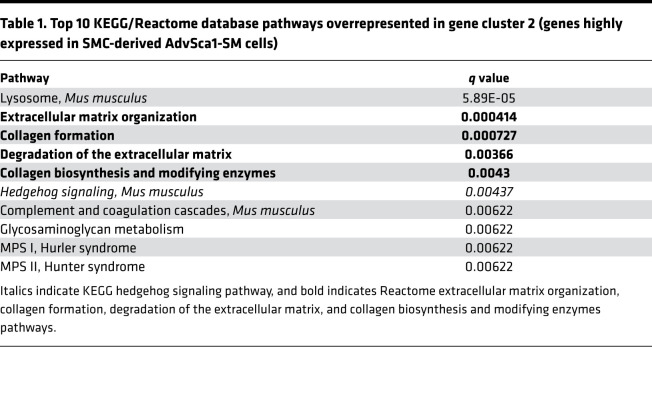
Top 10 KEGG/Reactome database pathways overrepresented in gene cluster 2 (genes highly expressed in SMC-derived AdvSca1-SM cells)

## Version 1 10/29/2020

In-Press Preview

## Version 2 12/03/2020

Electronic publication
