# Supplementary material for: Smooth muscle–derived progenitor cell myofibroblast differentiation through KLF4 downregulation promotes arterial remodeling and fibrosis
Source: JCI Insight. 2020 Dec 3;5(23):e139445. doi: 10.1172/jci.insight.139445 (PMC7714399; doi:10.1172/jci.insight.139445)
Supplement: supplemental data [file jciinsight-5-139445-s057.pdf]

SUPPLEMENTAL TABLE, FIGURES, AND FIGURE LEGENDS

Supplemental Table 1. qPCR primer sequences

Gene	Forward Primer	Reverse Primer
Gli1	5’-TCTGCGTGGTAGAGGGAACT-3’	5’-GGAGACAGCATGGCTCACTAT-3’
Gli2	5’-AAAAAGGCCTGTCCTCTGGC-3’	5’-TCCCTCCTGGTGTCTCATGT-3’
Wnt1	5’-CGAGTGTCTATGAGGTGCCG-3’	5’-GGTAGGGTGGGCAAACATCA-3’
Wnt2	5’-CTCGGTGGAATCTGGCTCTG-3’	5’-CACATTGTCACACATCACCCT-3’
Wnt5a	5’-GAATTCCTCGGCCGCCTTC-3’	5’-AACTTGGAAGACATGGCACCT-3’
Wnt9a	5’-GGTGGGCAAGCACCTAAAAC-3’	5’-GTACAAGCTCTGGTGTTCGGG-3’
Col1a1	5’-GCTCCTCTTAGGGGCCACT-3’	5’-CCACGTCTCACCATTGGGG-3’
Col3a1	5’-CTGTAACATGGAAACTGGGGAAA-3’	5’-CCATAGCTGAACTGAAAACCACC-3’
Col6a1	5’-CTACACCGACTGCGCCATTA-3’	5’-CCCCGCATGGTTCCTTGTAG-3’
Vcan	5’-AGCAGTGAATTTCCACCCCG-3’	5’-GGCTGGTTTCCATTTTGGCTT-3’
Dcn	5’-TTGATGCACCCAGCCTGAAA-3’	5’-GCCAGACTGCCATTCTCCAT-3’
Cd44	5’-TAGGAGAAGGTGTGGGCAGA-3’	5’-GGTTTCCTGTCTTCCACCGT-3’
Lox	5’-ACGCTGTGACATTCGCTACA-3’	5’-TGTCCAAACACCAGGTACGG-3’
Pcolce	5’-GCCCAAAGTCAAACCACCCA-3’	5’-GTGCCTGACCGCTTGTACT-3’
Bst1	5’-AGGGACAAGTCACTGTTCTGG-3’	5’-AACTTTGCCATACAGCACGTC-3’
Sema3g	5’-GGTTCCCTAGACCTCCAAGTC-3’	5’-GTCTTTTCCCTTGCGGACACA-3’
Mycn	5’-CCTCACTCCTAATCCGGTCAT-3’	5’-GTGCTGTAGTTTTTCGTTCACTG-3’
Itpripl1	5’-CGTGCCCGTCTCCTCTTAC-3’	5’-GTCACGGATGGCATTTTGGATA-3’
Lsr	5’-AGTGACACTACACTGCACCTA-3’	5’-CCCGACAGAACGACTTATACTTC-3’
Efr3b	5’-CGCCTGAAAAACTGGATCGAA-3’	5’-GGCAATACACACGTACCCATATC-3’
Igfbp3	5’-CCAGGAAACATCAGTGAGTCC-3’	5’-GGATGGAACTTGGAATCGGTCA-3’
Ly6c1	5’-GCAGTGCTACGAGTGCTATGG-3’	5’-ACTGACGGGTCTTTAGTTTCCTT-3’

Abcg2	5'-GAACTCCAGAGCCGTTAGGAC-3'	5'-CAGAATAGCATTAAGGCCAGGTT-3'
Abcb1a	5'-CAGCAGTCAGTGTGCTTACAA-3'	5'-ATGGCTCTTTTATCGGCCTCA-3'
β-Actin	5'-AGGGTGTGATGGTGGGTATGG-3'	5'-CGTTGACCTTAGTACCCAGGA-3'

Supplemental Table 2. Gene Ontology analysis (Downregulated genes in AdvSca1-SM cells from injured carotid artery).

**Go biological process**	**Fold enrichment**	**FDR**
Regulation of mesenchymal stem cell differentiation (GO:2000739)	8.38	3.49E-02
Intraciliary retrograde transport (GO:0035721)	7.54	1.40E-03
Metanephric glomerulus development (GO:0072224)	7.54	1.93E-02
Cell proliferation involved in kidney development (GO: 00772111)	7.04	1.06E-02
Renal system pattern specification (GO:0072048)	6.7	2.78E-02
Pattern specification involved in kidney development (GO:0061004)	6.7	2.78E-02
Segment specification (GO:0007379)	6.03	4.22E-03
Embryonic body morphogenesis (GO:0010172)	6.03	3.84E-02
Positive regulation of vascular endothelial growth factor signaling pathway (GO:0030949)	5.86	2.06E-02
Mesenchymal cell proliferation (GO:0010463)	5.65	5.84E-03
Regulation of glial cell apoptotic process (GO:0034350)	5.41	2.78E-02
Glomerulus vasculature development (GO:0072012)	5.32	7.86E-03
Postsynapse assembly (GO:0099068)	5.32	7.84E-03
Ureter development (GO:0072189)	5.29	4.12E-03
Positive regulation of cardiac muscle cell proliferation (GO:0060045)	5.21	3.30E-04
Metanephric nephron development (GO:0072210)	5.03	2.32E-04
Cell differentiation involved in metanephros development (GO:0072202)	5.03	3.62E-02
Positive regulation of non-canonical Wnt signaling pathway (GO:2000052)	5.03	3.62E-02
Kidney vasculature development (GO:0061440)	5.03	1.04E-02
Renal system vasculature development (GO:0061437)	5.03	1.04E-02

Supplemental Table 3. Gene Ontology analysis (Upregulated genes in AdvSca1-SM cells from injured carotid artery).

**Go biological process**	**Fold enrichment**	**FDR**
Toll-like receptor 7 signaling pathway (GO:0034154)	10.87	9.59E-03
Cytokine secretion involved in immune response (GO:0002374)	10.87	3.23E-03
Cellular response to diacyl bacterial lipopeptide (GO:0071726)	10.87	2.81E-02
Response to diacyl bacterial lipopeptide (GO:0071724)	10.87	2.81E-02
Positive regulation of type III hypersensitivity (GO:0001805)	10.87	2.81E-02
Regulation of type III hypersensitivity (GO:0001803)	10.87	2.81E-02
Regulation of immunological synapse formation (GO:2000520)	10.87	2.81E-02
Positive regulation of T-helper 17 cell differentiation (GO:2000321)	10.87	9.59E-03
Detection of lipopolysaccharide (GO:0032497)	10.87	2.81E-02
Negative regulation of myeloid dendritic cell activation (GO:0030886)	10.87	2.80E-02
Negative regulation of mature B cell apoptotic process (GO:0002906)	10.87	9.59E-03
Regulation of mature B cell apoptotic process (GO:0002905)	10.87	9.57E-03
Wound healing involved in inflammatory response (GO:0002246)	9.51	1.73E-03
Regulation of macrophage inflammatory protein 1 alpha production (GO:0071640)	9.06	1.51E-02
Cellular response to bacterial lipopeptide (GO:0071221)	9.06	1.51E-02
Cellular response to bacterial lipopeptide (GO:0071220)	9.06	1.51E-02
Mitotic spindle midzone assembly (GO:0051256)	9.06	1.51E-02
Response to bacterial lipopeptide (GO:0070339)	9.06	1.51E-02
Mititic spindle elongation (GO0000022)	9.06	1.51E-02
Negative regulation of T-helper 1 type immune response (GO:0002826)	9.06	1.51E-02

Supplemental Table 4. KEGG Pathway analysis (Downregulated genes in AdvSca1-SM cells from injured carotid artery).

KEGG pathways	Gene Ratio	FDR
Mmu05200:Pathways in cancer	3.75381929	2.24E-11
Mmu04151:PI3K-Akt signaling pathway	2.57529463	0.0071602
Mmu0415:Rap1 signaling pathway	2.00785683	9.52E-05
Mmu04014:Ras signaling pathway	1.92055871	0.0046917
Mmu05205:Proteoglycans in cancer	1.87690965	4.19E-04
Mmu04360:Axon guidance	1.74596246	9.54E-09
Mmu04519:Focal adhesion	1.74596246	0.0122987
Mmu04390:Hippo signaling pathway	1.61501528	7.30E-05
Mmu04550:signaling pathways regulating pluripotency of stem cells	1.52771715	7.24E-05
Mmu04921:Oxytocin signaling pathway	1.52771715	6.21E-04
Mmu04724:Glutamatergic synapse	1.48406809	1.83E-06
Mmu04310:Wnt signaling pathway	1.48406809	4.17E-04
Mmu04022:cGMP-PKG signaling pathway	1.44041903	0.0331967
Mmu04713:Circadian entrainment	1.35312091	1.88E-06
Mmu04723:Retrograde endocannabinoid signaling	1.22217372	4.50E-04
Mmu04916:Melanogenesis	1.04757748	0.0291947
Mmu05217:Basal cell carcinoma	0.96027935	4.57E-06
Mmu00982:Drug metabolism – cytochrome P450	0.96027935	2.70E-04
Mmu05414:Dilated cardiomyopathy	0.96027935	0.0164882
Mmu04540:Gap junction	0.96027935	0.0296349

Supplemental Table 5. KEGG Pathway analysis (Upregulated genes in AdvSca1-SM cells from injured carotid artery).

KEGG pathways	Gene Ratio	FDR
Mmu04060:Cytokine-cytokine receptor interaction	3.6596958	1.15E-11
Mmu05152:Tuberculosis	3.1844106	2.67E-14
Mmu04062:Chemokine signaling pathway	3.0893536	2.23E-10
Mmu05203:Viral carcinogenesis	2.756654	5.02E-04
Mmu05164:Influenza A	2.6140684	7.92E-08
Mmu04145:Phagosome	2.5665399	2.61E-07
Mmu04380:Osteoclast differentiation	2.5190114	8.77E-13
Mmu05168:Herpes simplex infection	2.4239544	0.0058830
Mmu05322:Systemic lupus erythematosus	2.2813688	8.92E-07
Mmu05202:Transcriptional misregulation in cancer	2.1863118	4.34E-04
Mmu05162:Measles	2.1387833	2.07E-06
Mmu04515:Cell adhesion molecules (CAMs)	1.9486692	0.0271406
Mmu05161:Hepatitis B	1.8536122	0.0119257
Mmu04620:Toll-like receptor signaling pathway	1.7585551	3.46E-06
Mmu04668:TNF signaling pathway	1.7585551	3.51E-05
Mmu05323:Rheumatoid arthritis	1.7110266	1.62E-08
Mmu05145:Toxoplamosis	1.7110266	4.21E-05
Mmu04640:Hematopoietic cell lineage	1.6634981	2.72E-07
Mmu04142:Lysosome	1.6634981	0.0070902
Mmu05140:Leishmaniasis	1.6159696	8.18E-11

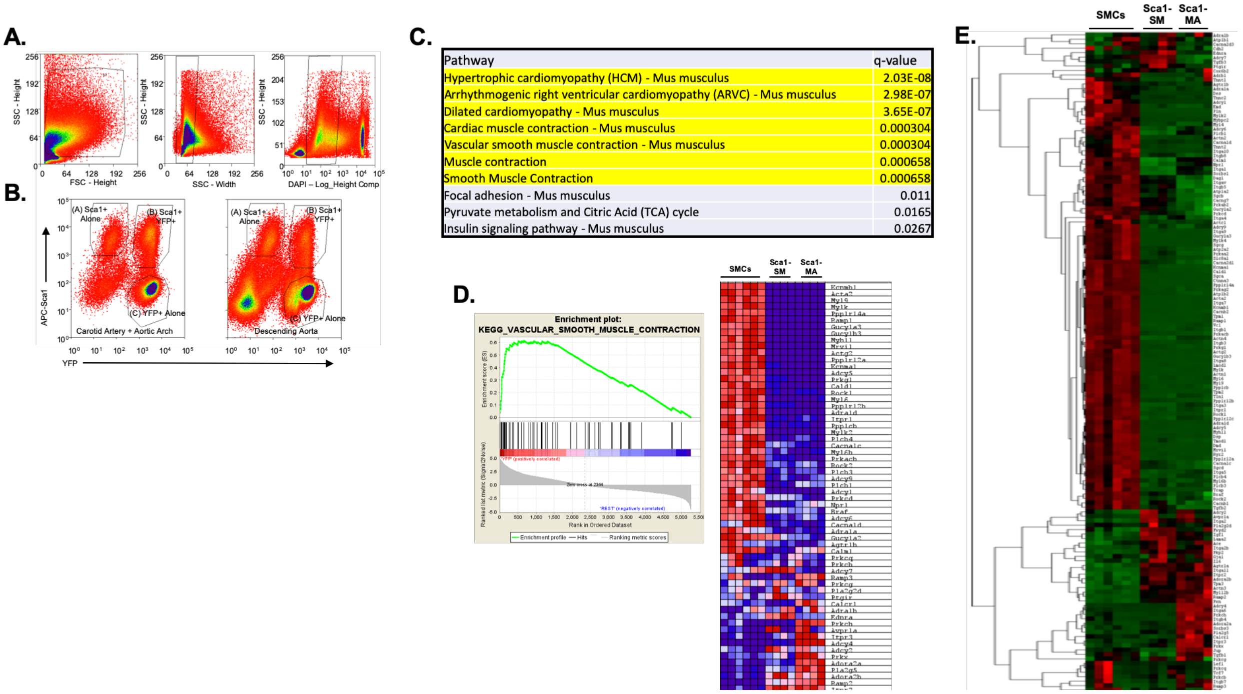

Supplemental Figure 1. Gating strategy for mature SMCs, SMC-derived AdvSca1-SM cells and non-SMC-derived AdvSca1-MA cells and SMC-specific and contractile genes are selectively expressed by mature SMCs. Carotid arteries plus aortic arch and descending aorta were harvested from two-month old SMC reporter mice. Arteries from 10-12 mice were pooled, digested into single cell suspensions, labeled with an APC-conjugated anti-Sca1 antibody, and flow sorted based on endogenous YFP and Sca1 expression. **(A).** Gating was performed on single cell suspensions based on forward and side scatter (left) followed by identification of singlets (middle). Single cells were plotted for DAPI to gate out dead cells (right). **(B).** Representative density plots showing three distinct cell populations: (A): YFP(-)Sca1(+) non-SMC-derived AdvSca1-MA cells, (B):YFP(+)Sca1(+) SMC-derived AdvSca1-SM cells, and C: YFP(+)Sca1(-) mature SMCs. **(C).** Top ten KEGG/Reactome database pathways overrepresented in gene Cluster 1 (genes highly expressed in mature SMCs). **(D).** Gene Set Enrichment Analysis (GSEA) enrichment plot (left) and KEGG vascular smooth muscle contraction heatmap (right). GSEA analysis tool was used to examine gene enrichment in mature SMCs compared to AdvSca1-SM and AdvSca1-MA cells. Red=upregulated genes; blue=downregulated genes. **(E).** Heatmap of levels of differentially expressed genes related to muscle contraction. Gene list was based on consensus of genes in the top-ranking muscle contraction pathways (KEGG: hypertrophic cardiomyopathy, arrhythmogenic right ventricular cardiomyopathy, dilated cardiomyopathy, cardiac muscle contraction, vascular smooth muscle contraction; Reactome: muscle contraction, smooth muscle contraction; yellow highlighted pathways in panel “C”). Red=upregulated genes; green=downregulated genes.

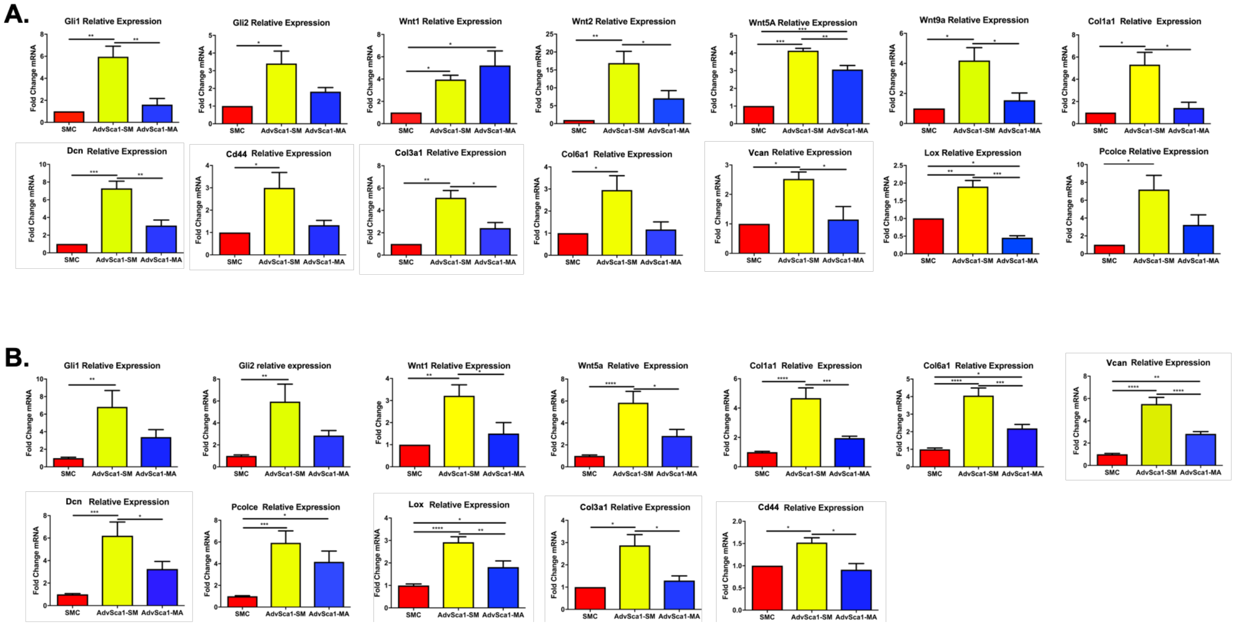

Supplemental Figure 2. qPCR validation of genes selectively upregulated in SMC-derived AdvSca1-SM cells. Mature SMCs (red bars), AdvSca1-SM cells (yellow bars), and AdvSca1-MA cells (blue bars) were recovered from the carotid artery + aortic arch (CA+arch) **(A)** or from descending aortae (dAo) **(B)** of SMC reporter mice as described in the Materials and Methods. Total RNA was isolated from cell populations from pooled, digested arteries and analyzed by qPCR for the indicated mRNAs. Shown are fold changes in mRNA copy number±SE from an N=3 independent experiments using arteries from 10-12 pooled mice per experiment; *P<0.05; **P<0.01; ***P<0.001. GAPDH was used for normalization. Expression of representative genes related to hedgehog/Wnt signaling and representative genes related to ECM and ECM remodeling are shown.

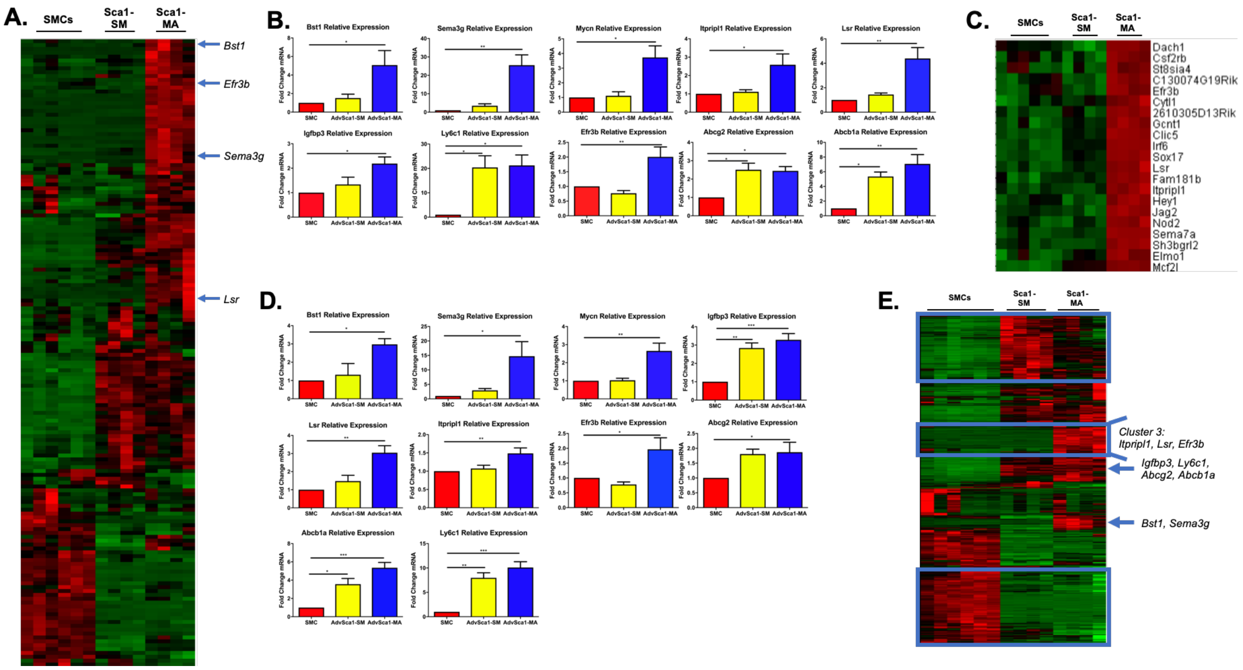

Supplemental Figure 3. Non-SMC-derived AdvSca1-MA cells express a tissue-resident endothelial stem cell gene signature. **(A).** Heatmap of all differentially expressed genes from Figure 1 compared to the top 500 upregulated genes in VESCs (1). The location of indicated genes selected for qPCR validation is shown on the right. **(B).** Mature SMCs (red bars), AdvSca1-SM cells (yellow bars), and AdvSca1-MA cells (blue bars) were recovered from the carotid artery + aortic arch (CA+arch), as described above. Total RNA was isolated from cell populations from pooled, digested arteries and analyzed by qPCR for the indicated mRNAs. Shown are fold changes in mRNA copy number±SE from an N=3 independent experiments using arteries from 10-12 pooled mice per experiment; **P*<0.05; ***P*<0.01. GAPDH was used for normalization. **(C).** Heatmap showing a comparison of the gene signature within cluster 3 (Figure 1) to the top 500 upregulated genes in VESCs (1). Heatmaps red=upregulated genes, green=downregulated genes. **(D).** Mature SMCs (red bars), AdvSca1-SM cells (yellow bars), and AdvSca1-MA cells (blue bars) were recovered from descending aortae (dAo) of SMC reporter mice as described in the Materials and Methods. Total RNA was isolated from cell populations from pooled, digested arteries and analyzed by qPCR for the indicated mRNAs. Shown are fold changes in mRNA copy number±SE from an N=3 independent experiments using arteries from 10-12 pooled mice per experiment; **P*<0.05; ***P*<0.01; ****P*<0.001; *P*<0.0001. GAPDH was used for normalization. **(E).** Location of indicated genes in heatmap shown in Figure 1 that were selected for qPCR validation, but not included in cluster 3.

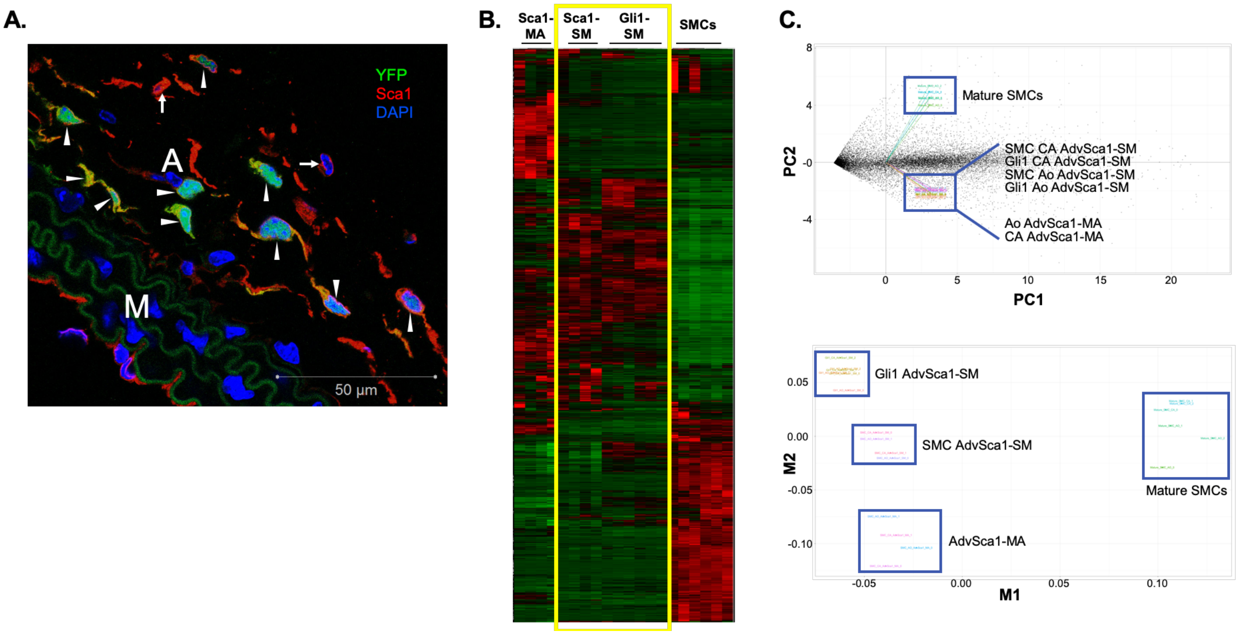

Supplemental Figure 4. **Gli1-Cre^{ERT}-YFP mice are selective AdvSca1-SM cell reporter mice. (A).** Representative carotid arterial section from Gli1-Cre^{ERT}-YFP reporter mice immunofluorescently stained for YFP (i.e. Gli1+ reporter; green) and Sca1 (red). Note YFP+ cells selectively reside in the arterial adventitia (A) and all YFP+ cells co-express Sca1 (arrowheads); not all Sca1+ adventitial cells co-express YFP (arrows). **(B).** RNA-Seq analysis of all differentially expressed genes between mature SMCs (right columns), AdvSca1-MA cells (left columns), and SMC reporter- and Gli1-YFP reporter-derived AdvSca1-SM cells (middle columns; boxed in yellow). Note the similar gene expression profile between SMC reporter- and Gli1-YFP reporter-derived AdvSca1-SM cells. **(C&D).** Principal component analysis (PCA; C) and multidimensional scaling (MDS; D) plots of normalized expression for the samples.

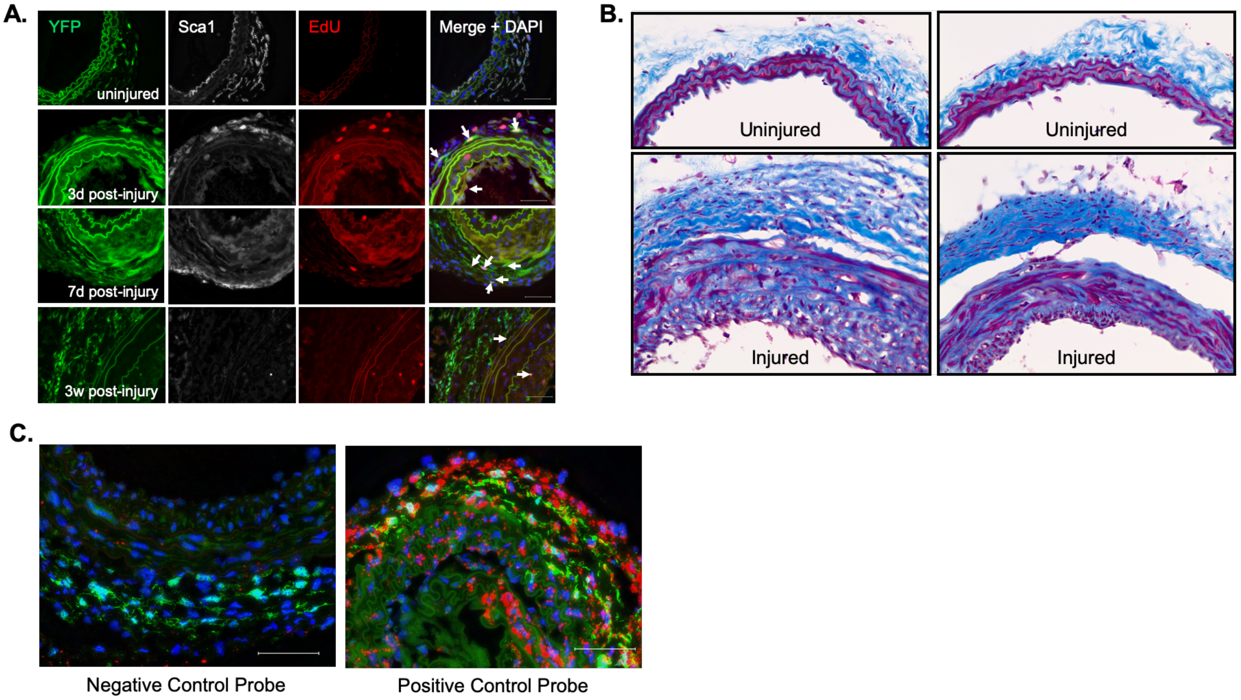

Supplemental Figure 5. Early proliferation of AdvSca1-SM cells in response to vascular injury, injury-induced vascular fibrosis, and negative and positive controls for periostin ISH. **(A).** Gli1-Cre^{ERT}-YFP mice were subjected to carotid arterial injury, mice were injected with EdU prior to sacrifice, and uninjured right and injured left carotids were harvested at 3d, 7d, and 3w post-injury. Arterial sections were immunofluorescently stained for YFP (green), Sca1 (white), and EdU (red). Representative images from N=5 (3d), N=10 (7d), and N=11 (3w). Scale bars = 50μm. M = arterial media; A = arterial adventitia, NI = neointima. Arrows indicate proliferating AdvSca1-SM cells. **(B).** Uninjured (top) and injured (bottom) carotid artery sections were stained with Masson's trichrome stain to detect of collagen deposition (blue). Shown are representative paired uninjured and injured arteries from two separate mice. **(C).** Carotid arterial sections from injured vessels were immunofluorescently stained for YFP (green) and *in situ* hybridization using a negative probe (DapB; top) or positive probe (Mm-Ppib; bottom; red) was conducted. Scale bars = 50μm.

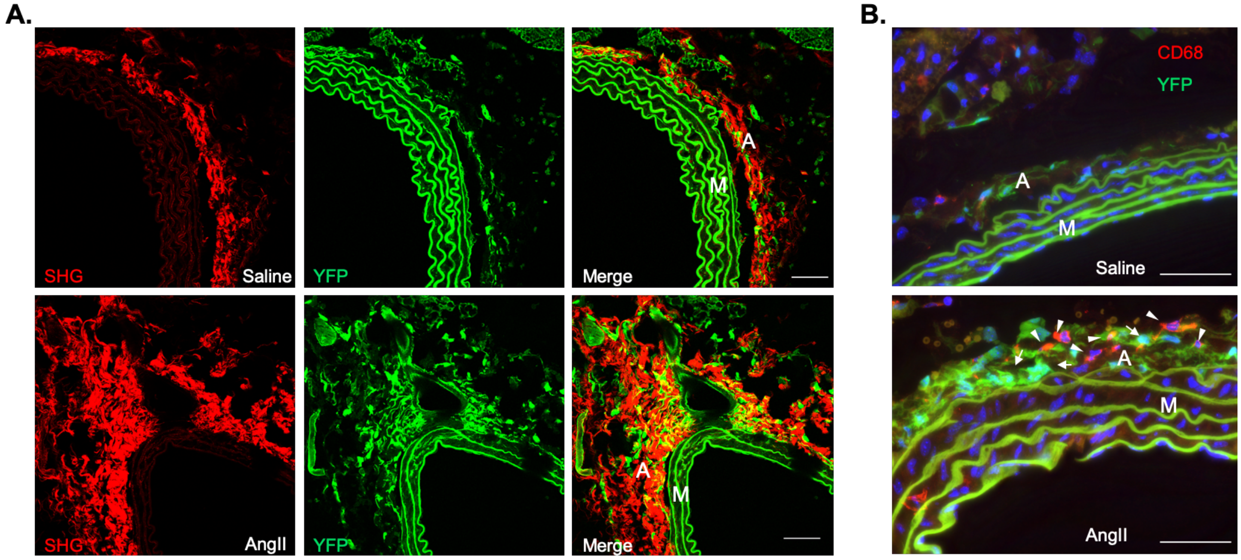

Supplemental Figure 6. Angiotensin II-mediated AdvSca1-SM cell contribution to adventitial fibrosis reveals rare AdvSca1-SM cell differentiation to macrophages. **(A).** Gli1-Cre^{ERT}-YFP mice treated with saline or Angiotensin II (1 µg/kg per min) for 28 d and aortic tissues were harvested. **(A).** Aortic sections were imaged for co-expression of YFP and label-free second harmonic generation for collagen deposition (red). Elastin autofluorescence is also observed on the green channel. **(B).** Aortic sections were immunofluorescently stained for YFP (green) and CD68 (red). Arrowheads=CD68(+) macrophages; arrows=CD68(-) AdvSca1-SM cells. Representative images from N=3 mice per condition. M=arterial media; A=arterial adventitia.

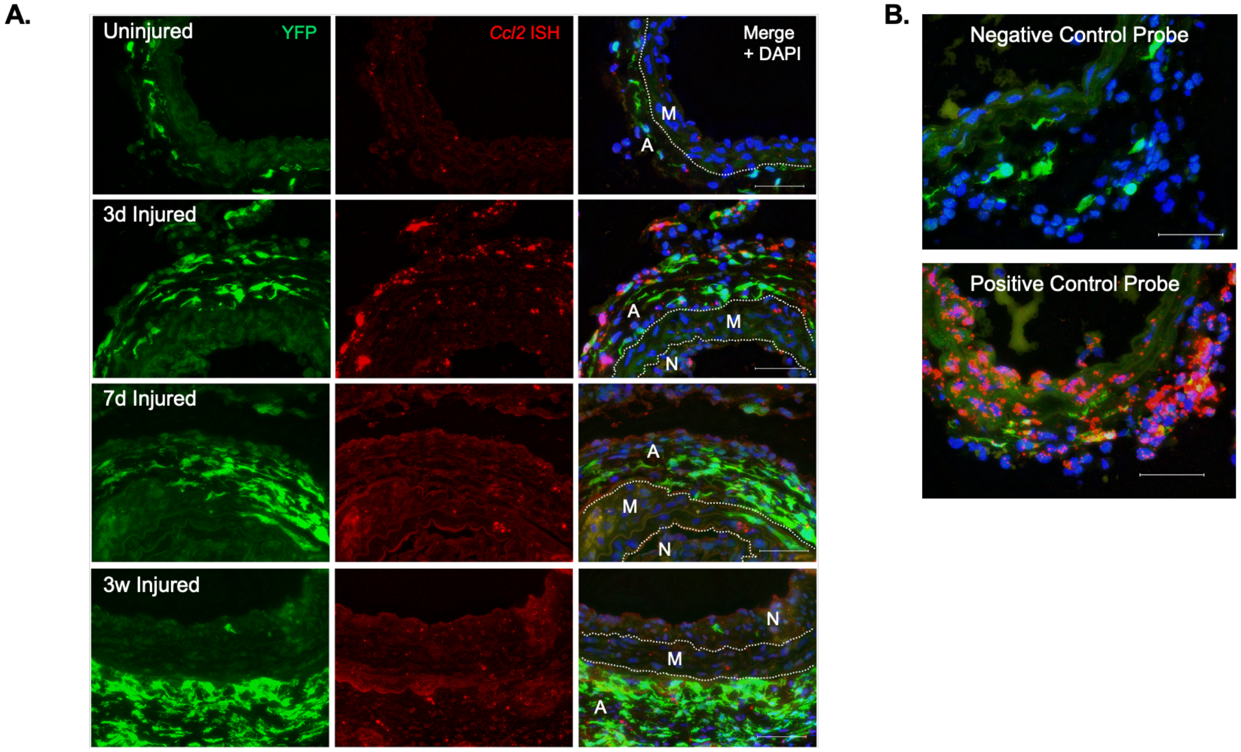

Supplemental Figure 7. Injury-mediated induction of Ccl2 in AdvSca1-SM cells. Gli1-Cre^{ERT}-YFP mice were subjected to carotid arterial injury and uninjured right and injured left carotids were harvested at 3d, 7d, and 3w post-injury, fixed, and embedded in OCT. **(A).** Arterial sections were immunofluorescently stained for YFP (green) and *in situ* hybridization was used to detect *Ccl2* transcripts (red). Representative images from N=3 at each time point. Note strong induction of *Ccl2* in AdvSca1-SM cells in response to injury. **(B).** Carotid arterial sections were immunofluorescently stained for YFP (green) and *in situ* hybridization using a negative probe (DapB; left) or positive probe (Mm-Ppib; right; red) was conducted.

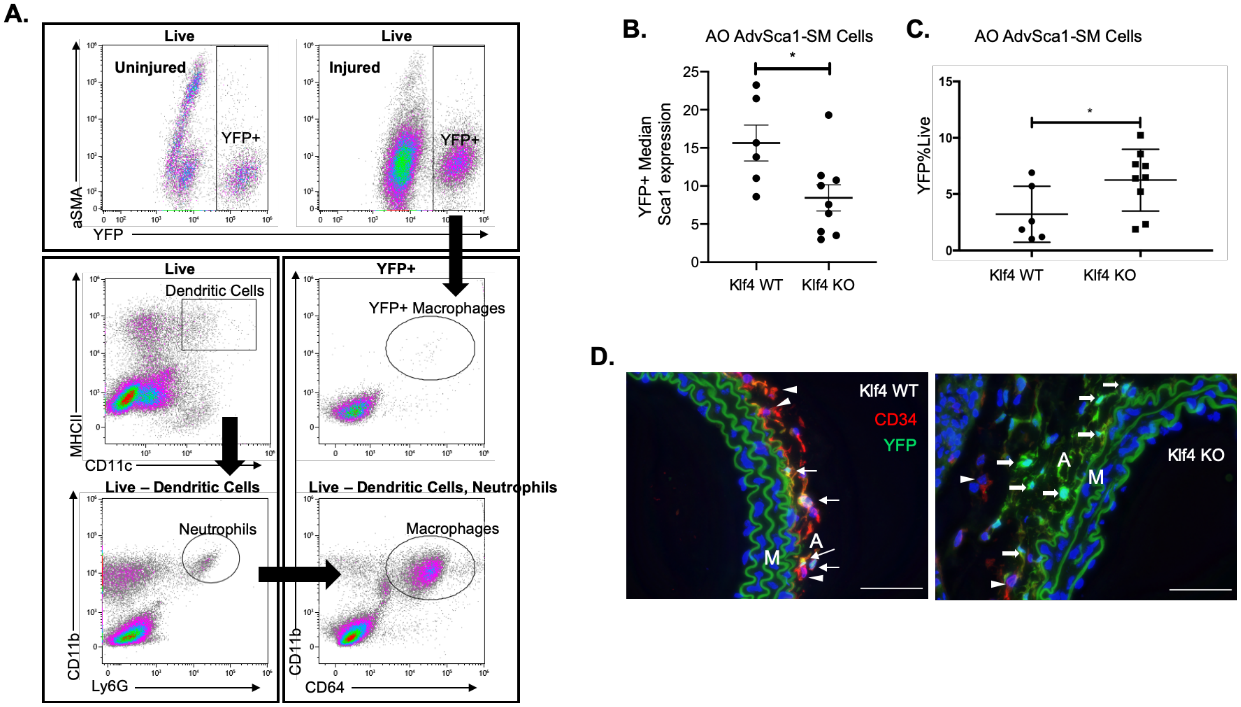

Supplemental Figure 8. Gating strategy for flow cytometry, spontaneous adventitial remodeling in AdvSca1-SM cell-specific Klf4 KO mice, and AdvSca1-SM cell loss of CD34 expression in AdvSca1-SM cell-specific Klf4 KO mice. **(A).** Flow cytometry was performed with single cell suspension prepared from uninjured and injured carotid artery. YFP and aSMA expression of live cells were examined with dot plot (top box). Macrophages were gated as $CD11b^{+}$ $CD64^{+}$ Live cells excluding $CD11c^{hi}$/$MHCII^{hi}$ dendritic cells and $CD11b^{hi}$/$Ly6G^{hi}$ neutrophils (bottom boxes). Macrophage populations from all live cells and YFP^{+} cells were examined in dot plot (bottom right box). Representative scatter plots from an injured carotid artery shown. **(B&C).** WT and Klf4 KO $Gli1Cre^{ERT}$-YFP mice were injected with tamoxifen as described in Materials and Methods. Descending aortic tissues were harvested 4 weeks after the final tamoxifen injection. Single cell suspensions were isolated, stained for Sca1, and analyzed by flow cytometry for quantification of Sca1 expression in YFP(+) AdvSCa1-SM cells **(B)** and for quantification of total YFP(+) AdvSca1-SM-derived cells **(C)**. Each point represents a single mouse; N=6 (WT) and N= 9 (KO). **(D).** Carotid artery sections were immunofluorescently stained for CD34 (red) and YFP (green). Arrows = YFP(+)CD34(+) AdvSca1-SM cells; Block arrows = YFP(+)CD34(-) AdvSca1-SM cell-derived cells; Arrowheads = YFP(-)CD34(+) AdvSca1-MA cells.

REFERENCES

1. Wakabayashi T, Naito H, Suehiro JI, Lin Y, Kawaji H, Iba T, Kouno T, Ishikawa-Kato S, Furuno M, Takara K, Muramatsu F, Weizhen J, Kidoya H, Ishihara K, Hayashizaki Y, Nishida K, Yoder MC, Takakura N. CD157 Marks Tissue-Resident Endothelial Stem Cells with Homeostatic and Regenerative Properties. *Cell Stem Cell.* 2018 Mar 1;22(3):384-397.e6.
